# A randomized controlled proof-of-concept trial of early sedation management using Responsiveness Index monitoring in mechanically ventilated critically ill patients

**DOI:** 10.1186/s13054-015-1043-1

**Published:** 2015-09-11

**Authors:** Markus Kaila, Kirsty Everingham, Petteri Lapinlampi, Petra Peltola, Mika O K Särkelä, Kimmo Uutela, Timothy S. Walsh

**Affiliations:** Anaesthetics, Critical Care and Pain Medicine, Centre for Inflammation Research and School of Clinical Sciences, Edinburgh University, 51 Little France Crescent, Edinburgh, Scotland EH16 4SA UK; GE Healthcare Finland Oy, Kuortaneenkatu 2, 00510 Helsinki, Finland

## Abstract

**Introduction:**

Deep sedation is associated with adverse patient outcomes. We recently described a novel sedation-monitoring technology, the Responsiveness Index (RI), which quantifies patient arousal using processed frontal facial EMG data. We explored the potential effectiveness and safety of continuous RI monitoring during early intensive care unit (ICU) care as a nurse decision-support tool.

**Methods:**

In a parallel-group controlled single centre proof of concept trial, patients requiring mechanical ventilation and sedation were randomized via sequential sealed envelopes following ICU admission. Control group patients received hourly clinical sedation assessment and daily sedation holds; the RI monitor was connected but data were concealed from clinical staff. The intervention group received control group care, but RI monitoring was visible and nurses were asked to adjust sedation to maintain patients with an RI>20 whenever possible. Traffic-light colour coding (RI<20, Red; 20–40, Amber; >40, Green) simplified decision-making. The intervention lasted up to 48 hours. Sixteen nurses were interviewed to explore their views of the novel technology.

**Results:**

We analysed 74 patients treated per protocol (36 intervention; 38 control). The proportion of patients with RI<20 was identical at the start of monitoring (54 % both groups). Overall, the proportion of time with RI<20 trended to lower values for the intervention group (median 16 % (1–3rd quartile 8–30 %) versus 33 % (10–54 %); P = 0.08); sedation and analgesic use was similar. A *post hoc* analysis restricted to patients with RI<20 when monitoring started, found intervention patients spent less time with low RI value (16 % (11–45 %) versus 51 % (33–72 %); P = 0.02), cumulative propofol use trended to lower values (median 1090 mg versus 2390 mg; P = 0.14), and cumulative alfentanil use was lower (21.2 mg versus 32.3 mg; P = 0.01). RASS scores were similar for both groups. Sedation related adverse event rates were similar (7/36 versus 5/38). Similar proportions of patients had sedation holds (83 % versus 87 %) and were extubated (47 % versus 44 %) during the intervention period. Nurses valued the objective visible data trends and simple colour prompts, and found RI monitoring a useful adjunct to existing practice.

**Conclusions:**

RI monitoring was safe and acceptable. Data suggested potential to modify sedation decision-making. Larger trials are justified to explore effects on patient-centred outcomes.

**Trial registration:**

NCT01361230 (registered April 19, 2010)

**Electronic supplementary material:**

The online version of this article (doi:10.1186/s13054-015-1043-1) contains supplementary material, which is available to authorized users.

## Introduction

Most mechanically ventilated critically ill patients require sedation and analgesia. Deep sedation is associated with adverse outcomes including higher mortality [1, 2], and strategies that systematically avoid over-sedation improve patient outcomes in both randomised trials [3–6] and quality improvement studies [7–9]. Conversely, light sedation and agitation can compromise patient safety and increase staff workload and stress [10–12].

Recent guidelines recommend the systematic evaluation of pain, agitation, and delirium in intensive care units (ICUs) and implementation of evidence-based strategies to improve patient experience and outcomes [13, 14]. Although there are valid and reliable clinical tools for diagnosing and rating pain, sedation and delirium [15], the implementation of practices that identify and systematically avoid unnecessary deep sedation is challenging. There are several technologies for monitoring sedation status based on electroencephalogram (EEG) analysis, but these were developed primarily for monitoring the depth of anaesthesia and their validity for use in ICU patients is uncertain [16]. Although these algorithms correlate with clinical sedation scores, their discriminant ability is limited, in part because of interference from facial frontal electromyelogram (fEMG) signals during arousals and in lighter sedation states [17, 18]. There have been few randomised trials of these technologies, and the available data do not support clinical effectiveness [13, 19].

We recently described a novel technology for continuous monitoring of patient status in ICU patients, the Responsiveness Index (RI), based on fEMG activity [20–22]. The algorithm uses fEMG data acquired via adhesive surface forehead electrodes, and utilises the previous 60 minutes of fEMG activity to assess the frequency and intensity of arousals during ongoing treatments. RI has been shown to perform better than an EEG-based algorithm [21], and to have face and criterion validity compared with clinical sedation states [20].

Our aim in this proof-of-concept trial was to assess the effectiveness, safety, and acceptability of continuous RI monitoring during early ICU care as a nurse decision-support tool. We hypothesised that continuous RI monitoring as an adjunct to clinical sedation scoring would decrease the period of low patient responsiveness without excess adverse events, compared to a control group treated according to current best practice. We aimed to use both quantitative and qualitative approaches to provide early effectiveness, safety and acceptability data.

## Methods

We undertook a prospective single-centre randomised parallel-group controlled proof-of-concept trial, including qualitative analysis of interviews with nursing staff utilising the RI monitor.

### Patients and setting

The study took place in an 18-bed Scottish general adult ICU admitting approximately 650 ventilated medical, surgical, and trauma patients annually (excluding routine cardiac surgery and neuro-intensive care). Patients were screened by clinical nursing staff at the time of ICU admission. Inclusion criteria were that patients were mechanically ventilated via an endotracheal tube, and receiving intravenous sedation with a hypnotic agent by continuous infusion. Exclusion criteria were patients who had a primary intracerebral disorder (including post-cardiac arrest, intracranial haemorrhage, or head injury causing a reduced level of consciousness prior to intubation); were already conscious at the time of enrolment defined as Richmond agitation-sedation scale (RASS) score [23] ≥ −1; were aged <16 years; were not expected to survive the next 24 hours; were receiving long-term ventilation prior to ICU admission; had a long-term tracheostomy prior to ICU admission; had been transferred sedated and mechanically ventilated from another ICU (unless recruitment was possible within 24 hours of the first ICU admission); were receiving continuous neuromuscular blocking agent (NMBA) at the time of screening; had previously been enroled in the trial; had status epilepticus; had confirmed meningitis or encephalitis, or had a known chronic neurological disease interfering with normal neuromuscular function, e.g., motor neurone disease, Guillain-Barre syndrome or inherited neuromyopathy.

### Consent and randomization

Consent was deferred to enable the establishment of responsiveness monitoring close to the time of ICU admission. Relatives or next of kin were approached at the earliest opportunity following admission for consent to remain in the study; patients were approached when they had capacity. Patients in whom consent was not subsequently obtained from a relative, or patients who were withdrawn from the trial were excluded from the analysis (according to Scottish legal requirements). Patients were randomised by nursing staff to one of the two groups by opening sequentially numbered opaque sealed envelopes. The randomisation order was generated using a computer randomisation sequence. No stratification or minimisation was used. The allocation within each envelope directed the clinical nurse to select an RI monitor pre-specified to the control or intervention group (see below). The study was approved by the Scottish A Research Ethics committee (09/MRE00/17).

### Study conduct

All participating nursing staff received pre-trial training in the use of the Responsiveness monitor, and the concept that low RI values are expected for deeply sedated patients, for some patients in a coma unrelated to sedation, and during natural sleep [20]. Nurses were also instructed not to use monitor data when patients received NMBAs.

### Control group (group 1)

All patients were attached to the Responsiveness monitor, and data were acquired using a laptop computer. For these patients a monitoring device was used, which did not display any RI data throughout the study. RI data could only be accessed by code by the research team (to check quality etc.), which was not available to clinical staff. The monitor presented an automated prompt every hour during the study to assess the RASS score and enter it into the study case record form (CRF). The prompt flashed on the monitor until a score was entered, and the exact time of entry logged. The local sedation protocol encouraged staff to target a RASS score of >−3 and to undertake a sedation hold on a daily basis unless clinically contraindicated.

### Intervention group (group 2)

**Fig. 1:**
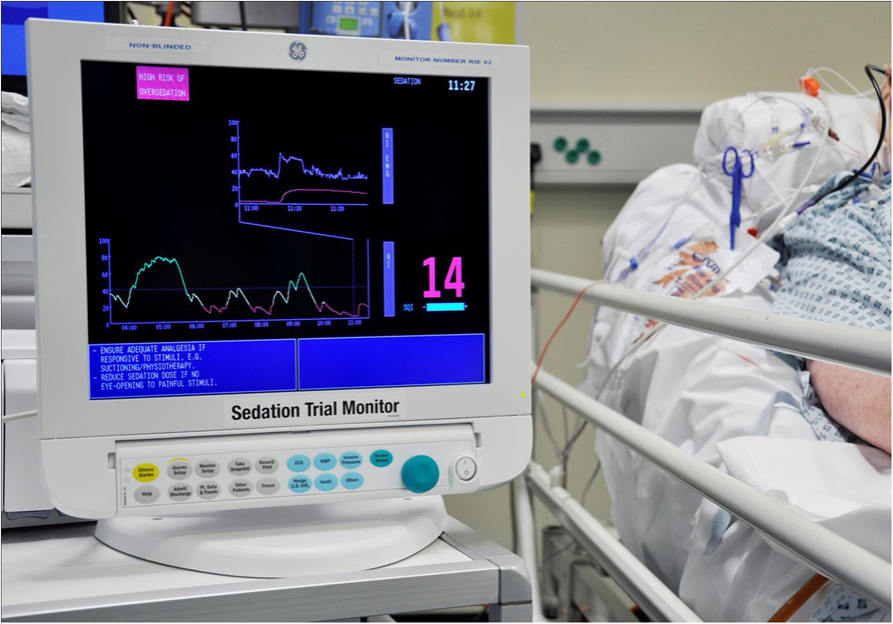
Responsiveness Index (RI) sedation monitor used in the trial. For intervention patients, the screen presented a running trend over several hours, with the most recent value presented on an arbitrary scale of 0–100, with a colour code of *RED* (<20), *AMBER* (20 to 40); and *GREEN* (>40). In addition to the colour trend, the following prompts were automatically presented according to RI value: *RED* high risk of over-sedation. Ensure adequate analgesia if responsive to stimuli, e.g., suctioning/physiotherapy. Reduce sedation dose if no eye-opening to physical stimuli. *AMBER*: moderate risk of over-sedation. Ensure adequate analgesia. Reduce sedation if no eye-opening to physical stimuli. *GREEN*: low risk of over-sedation. Ensure adequate analgesia. Continue current sedation unless patient agitated

As for group 1, all patients in the intervention group were attached to the Responsiveness monitor, and data collected using a laptop computer. For these patients RI data were presented as a continuous trend, with a numeric value (0–100), and a colour code (red, amber, green) according to our previous published work, which suggested a value with a red colour had sensitivity to detect patients who may be excessively sedated [20]. An example of a monitor during use is shown in Fig. 1. Prompts accompanied the colour of RI presented on the screen, to encourage sedation reduction for patients with a red or amber RI value (RI <40). Nurses were asked to alter sedation using clinical judgement to transition patients out of the red RI range and adjust sedation to achieve values in the amber/green range. In addition, automated prompts occurred every hour for RASS scores as for group 1.

### Management during intervention period

The choice of sedative and analgesic drugs, and the initial dosage, was at the discretion of the clinical team. The standard sedative drug was propofol, and the opiate alfentanil at the time of the study. Clinicians could use alternative agents, principally midazolam and morphine, according to clinical discretion. All decisions about other aspects of usual care were made by the clinical team, including the timing of extubation. The only trial-specific interventions were related to changes in the dose of sedatives made by bedside nursing staff in the two study groups. The research team, who were the only individuals with access to the RI data for the control group, was not involved in clinical management.

### Study endpoints

Responsiveness monitoring was continued until one of the following endpoints occurred:

1. 48 hours had elapsed from ICU admission
2. The patient was extubated
3. The patient died or a decision was made to withdraw treatment

The maximum duration of RI monitoring was 48 hours. This duration was chosen pragmatically for the proof-of-concept study, and to enable comparison for a fixed period of care during which excessive sedation is known to be associated with adverse outcomes [1].

### Discontinuing RI monitoring

RI monitoring could be discontinued for procedures or other reasons according to clinical judgement. In these cases, monitoring was reattached as soon as feasible if within the 48 hour intervention period.

### Data collection

Baseline data were age, gender, admission diagnosis, time from hospital to ICU admission, acute physiology and chronic health evaluation (APACHE) II score, Charlson comorbidity index, sequential organ failure assessment (SOFA) score (excluding neurological score) for the first 24 hours (range 0–20), RASS score at the start of monitoring, and intravenous sedation and analgesic drug use in ICU prior to RI monitoring. Hourly data collection during the intervention period was RASS score, and intravenous sedative and analgesic dose. The use of formal sedation holds was recorded on a daily basis. The following pre-defined sedation-related adverse events were recorded: unplanned extubation, unplanned removal of vascular catheter, unplanned removal of nasogastric/enteral tube, episode of myocardial ischaemia, myocardial infarction and agitation requiring pharmacologic treatment.

### Outcomes

Our feasibility outcomes were to test the protocol and determine likely recruitment rates for a large trial. To explore potential clinical effectiveness, acceptability, and safety we measured the following additional outcomes:

1. Proportion of time spent with low responsiveness (red colour code; RI <20) during the first 48 hours in ICU (or from when first sedated and ventilated)
2. Proportion of time spent with RASS score −4/−5 during first 48 hours (or from when first sedated and ventilated)
3. Sedation-related adverse event rates (comparison of control and intervention groups during intervention period)
4. Duration of mechanical ventilation
5. ICU, hospital mortality
6. Total sedative drug dose during first 48 hours in the ICU (or from when first sedated and ventilated)
7. Total opioid drug dose during first 48 hours in the ICU (or from when first sedated and ventilated)

We described patient status at 48 hours and 7 days post-randomisation. The full trial protocol is available in the additional material (see Additional file 1), and is registered at ClinicalTrials.gov: NCT01361230.

### Sample size

This was a proof-of-concept study so no formal sample size estimation was undertaken. We aimed to randomise 60–70 patients to assess feasibility and provide preliminary efficacy and safety data. Based on ICU admission rates (650 mechanically ventilated cases per year), and an estimated 20–30 % enrolment rate for eligible patients, we planned to recruit for a fixed period of 8 months.

### Analysis

The intervention period was defined as the start of monitoring to 48 hours post ICU admission. Baseline RI was defined as the first valid RI value recorded. For RI analyses patients in whom monitoring was removed because the patient was extubated were assumed to have an RI value >20 during the post-extubation period. Periods during which NMBAs were used, plus the 30 minutes after discontinuation, were censored from the analysis period. No deaths occurred during the 48-hour intervention period, so competing risk of death was not relevant.

RASS data were pre-processed to impute missing data using several rules: first, if RASS scoring was not performed during a particular hour, the subsequent hourly RASS value was utilized in the analysis; if the subsequent hour had no RASS value, the previous hourly value was used; if neither value was available then that hour was considered to have no RASS data point. For comparisons of total intravenous sedative and analgesic dose during the intervention periods propofol and alfentanil equivalents were calculated for patients who received midazolam and/or morphine as an alternative or addition to propofol/alfentanil. We used a conversion factor of 1 mg midazolam = 10 mg propofol, and 1 mg morphine = 100 μg alfentanil.

Prior to the trial we had no data to inform the distribution of RI values at enrolment. As the intervention aimed to promote transition from an RI value of <20 (red colour code) to higher RI values (amber/green colour code) we undertook a post hoc subgroup analysis for patients with baseline RI <20. We justified this analysis because for this group the protocol directed nursing staff to decrease sedation at enrolment, whereas for patients with RI values >20 the protocol did not direct nurses to alter sedation based on the intervention.

As this was a proof-of-concept study, we restricted analysis to patients treated per protocol with the monitor. We used the Wilcoxon rank-sum test to compare continuous non-parametric data, and Fisher’s exact test for categorical data. The log-rank test was used to compare time to first RI value >20, first RASS score >−4, and time to extubation. A *P* value <0.05 was considered statistically significant, but given the exploratory nature of the study we examined trends in addition to statistically significant differences.

### Qualitative study

Sixteen nurses with at least 12 hours experience using the monitor in the intervention arm were interviewed to explore their views of sedation management and the potential value of the RI technology. A phenomenological theoretical perspective was taken during analysis. The design of the qualitative study, underpinning theoretical perspective, and qualitative methods used are described in full in the additional material (see Additional file 2).

## Results

Ninety patients were randomised. No consent was obtained post-randomisation in 12 patients (data could not be utilised according to local ethics approval) (4 in the intervention group and 8 in the control group), a protocol deviation occurred in 4 patients (the randomisation procedure was incorrectly followed by clinical nursing staff in 2 patients, and monitoring was not started by clinical staff during sedation in 2 patients despite randomisation to the intervention group). We included 74 patients in the analysis (36 in the intervention group and 38 in the control group). Patient characteristics and baseline data are shown in Table 1. The patient groups were similar at baseline with respect to all measured variables. Monitoring was started within 8 hours of intubation for >75 % of participants.

**Table 1:** Baseline characteristics and patient status at randomisation

Baseline variables	Intervention (n = 36)	Control (n = 38)
Age, years, median	60	59
(1st, 3rd quartile; min–max)	(44, 69; 25–85)	(43, 72; 27–80)
Sex, male/female	21/15	26/12
Primary diagnosis, number of patients		
Cardiovascular	8	5
Respiratory	3	9
Gastrointestinal	14	15
Renal	0	1
Trauma	3	1
Other	8	7
APACHE II score, median	20	23
(1st, 3rd quartile; min–max)	(11, 24; 0–31)	(17, 26; 0–38)
Charlson comorbidity index, median	1	1
(1st, 3rd quartile; min–max)	(0, 2; 0–5)	(0, 2; 0–7)
SOFA score for first 24 hours, median	7	8
(1st, 3rd quartile; min–max)	(5, 11; 2–16)	(5, 9; 1–16)
Time, hours from intubation to start of monitoring, median	4.0	4.9
(1st, 3rd quartile; min–max)	(2.1, 6.2; 1.0–12.0)	(3.5, 7.7; 0.7– 1.8)
RI at start of intervention period, median	16	18
(1st, 3rd quartile; min–max)	(0, 55; 0–100)	(0, 42; 0–100)
Red, n (%)	19 (54.3 %)	20 (54.1 %)
Amber, n (%)	5 (14.3 %)	8 (21.6 %)
Green, n (%)	11 (31.4 %)	9 (24.3 %)
No value, n (%)^a^	1 (2.8 %)	1 (2.6 %)
RASS score at start of intervention period, median	−3	−4
(1st, 3rd quartile; min to max)	(−4, −2; −5 to +0)	(−4, −3; −5 to +2)
Total alfentanil dose, mg, prior to monitoring, median	1.75	3.00
(1st, 3rd quartile; min–max)	(0.75, 5.25; 0.00–14.00)	(1.00, 7.50; 0.00–17.50)

^a^One patient in each group had a long delay in commencing Responsiveness Index (*RI*) monitoring by clinical staff after randomisation. These two patients were not given a baseline RI value, and were excluded from the subgroup analysis of patients with RI <20 at baseline, as the value was uncertain at the start of the study. *APACHE* acute physiology and chronic health evaluation, *SOFA* sequential organ failure assessment, *RASS* Richmond agitation-sedation scale

During the RI monitoring period, NMBAs were administered to 10 patients (6 in the intervention group and 4 in the control group). These were mostly single bolus doses (n = 7); other durations were 30 minutes (n = 2) and 150 minutes (n = 1). The median percentage of time without monitoring was 2 % (1st, 3rd quartile: 0, 7 %) for the intervention and 3 % (0, 8 %) for the control group.

All intervention group patients and 36/38 control patients received propofol during the intervention period; 33/36 intervention group patients and 37/38 control patients received alfentanil. In the intervention group, 4/36 patients received midazolam as did 4/38 control group patients, and 8/36 patients in the intervention group and 7/38 control group patients received morphine during the intervention period. These were converted to propofol and alfentanil equivalents as described above.

**Fig. 2:**
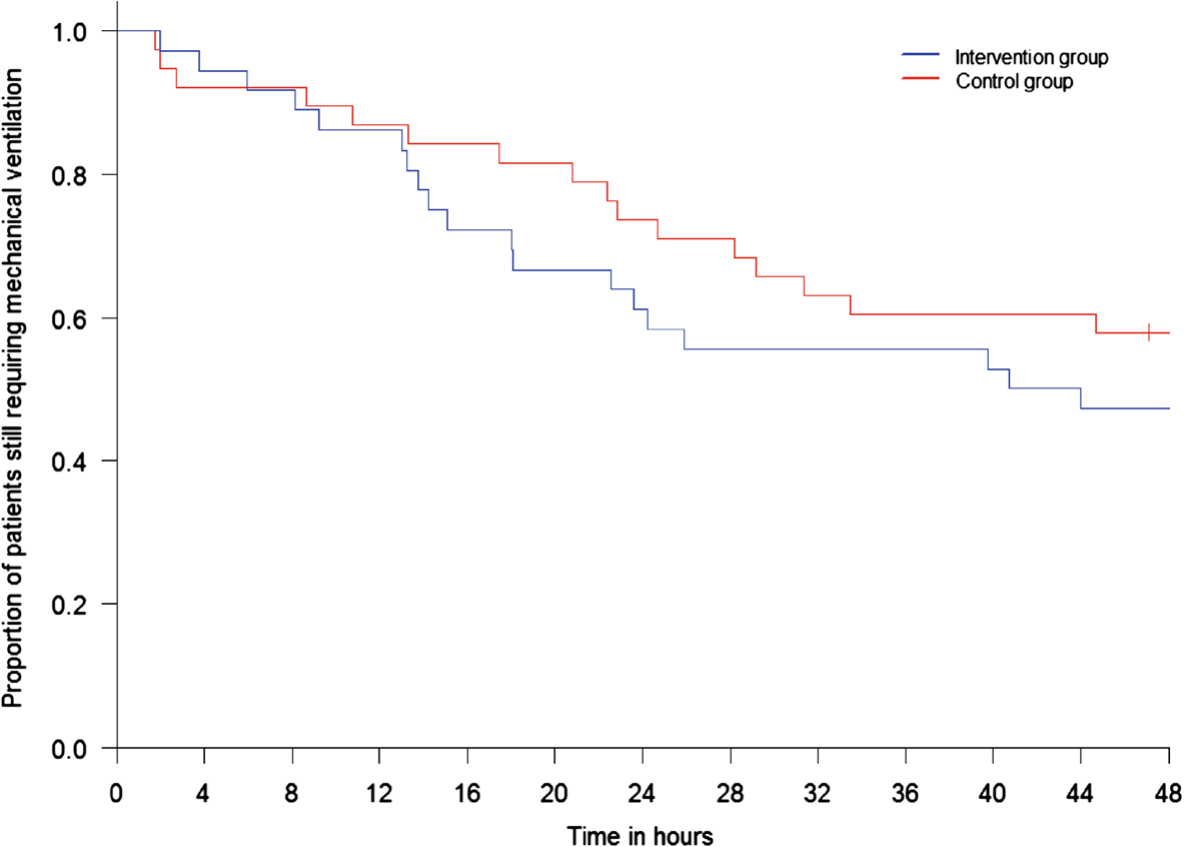
Kaplan-Meier curve illustrating the numbers of patients in each trial group (36 in the intervention and 38 in the control group) requiring mechanical ventilation over the 48-hour intervention period. Log-rank test: *P* = 0.33

**Fig. 3:**
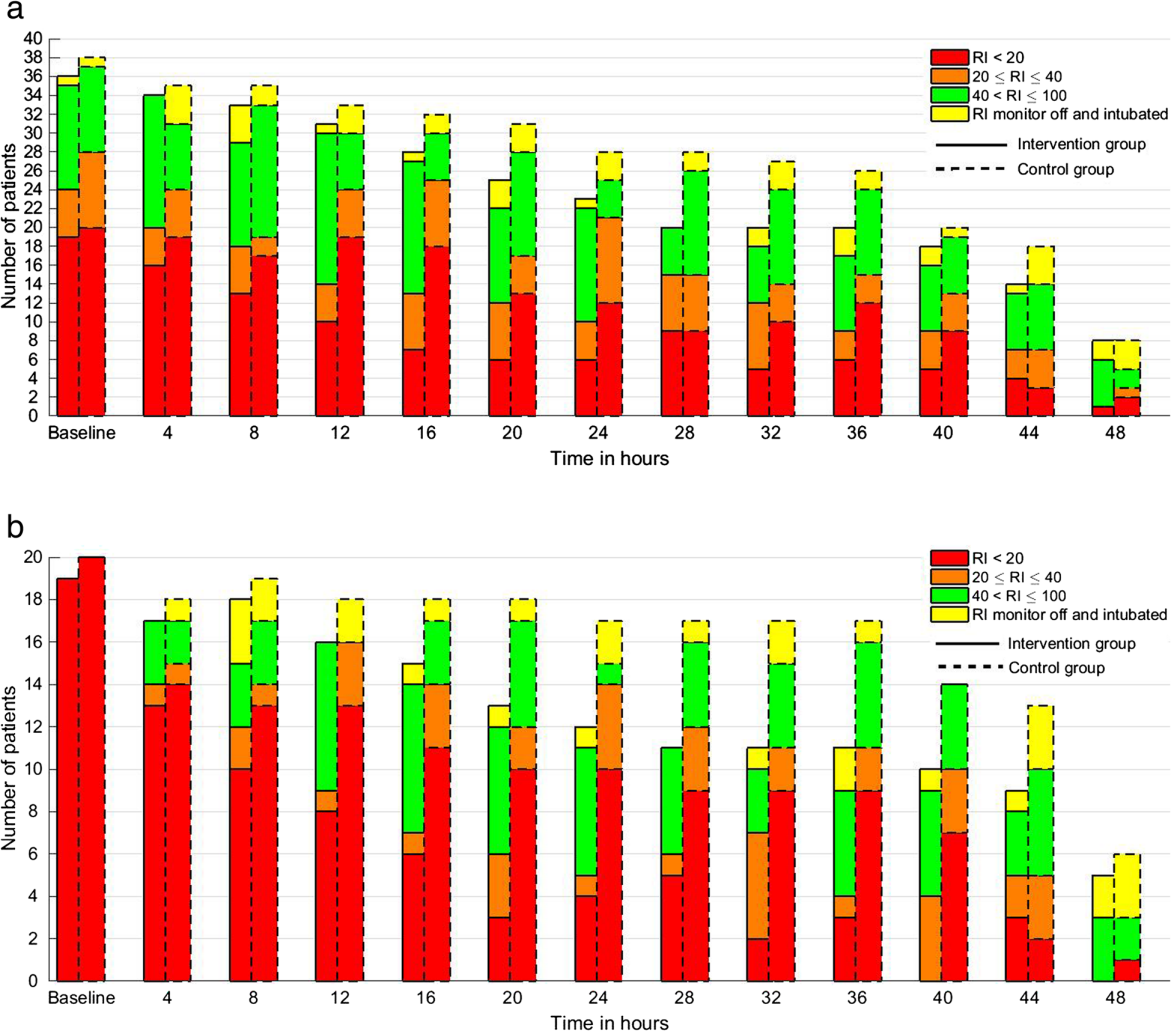
Description of the numbers of patients remaining intubated over the 48-hour intervention period, together with the numbers of patients with Responsiveness Index (*RI*) values in the *Red*, *Amber*, and *Green* categories at each time point. **a** Summary of data for for all patients in each group. **b** Data restricted to patients in whom the baseline RI value was <20

A comparison of outcomes between the groups is shown in Table 2. A high perentage of patients (>80 %) in both groups underwent a sedation hold during the intervention period, and 34/74 patients (46 %) were extubated. Among the entire cohort the intervention group patients tended to have fewer hours with RI <20, but this was not statistically significant (median 16 % (quartile 1–3, 8–30 %) versus 33 % (10–54 %); *P* = 0.08). Mechanical ventilation status over the 48-hour intervention period is shown in Fig. 2. There were no statistically significant differences between the groups overall, and no difference in sedation-related adverse events. In the post hoc analysis, 53 % of patients had a baseline RI of <20 (19 in the intervention group and 20 in the control group). Among these patients those in the intervention group spent less time with RI <20 (16 % (11–45) in the intervention versus 51 % (33–72) in the control group; *P* = 0.02), and also tended to receive lower cumulative doses of propofol (P = 0.14) and alfentanil (*P* = 0.01) (Table 3). Changes in ventilation and RI status for all patients over the 48 hour-intervention period are illustrated in Fig. 3a, and for the sub-group with RI <20 at baseline in Fig. 3b. These indicated a pattern of quicker extubation and higher RI values over time for the intervention group, especially when the baseline RI was <20.

**Table 2:** Comparison outcomes during the intervention period and at 7 days post-randomisation for the entire study cohort

Outcome	Intervention (n = 36)	Control (n = 38)	*P* value
Outcomes during 48-hour intervention period			
Proportion of hours during the intervention period with RI value <20, median	16	33	0.08
(1st, 3rd quartile; min– max)	(8, 30; 1–87)	(10, 54; 0–86)	
Time, hours from start of monitoring to first RI value >20, median	0.1	0.3	0.43
(1st, 3rd quartile; min– max)	(0.0, 2.2, 0.0–7.8)	(0.0, 3.1, 0.0–48)	
Proportion of RASS scores of −4 or −5 during the intervention period, median	12	17	0.70
(1st, 3rd quartile; min– max)	(0, 35; 0–94)	(3, 36; 0–100)	
Time, hours from start of monitoring to first RASS score >−4, median	2	3	0.68
(1st, 3rd quartile; min– max)	(0, 8, 0–43)	(0, 8, 0–48)	
Total propofol dose, mg, during the intervention period, median	1,365	1,730	0.64
(1st, 3rd quartile; min– max)	(380, 2,790; 50–8,710) 1730	(620, 3,260; 0–8,630)	
Total alfentanil dose, mg, during the intervention period, median	23.4	25.2	0.68
(1st, 3rd quartile; min– max)	(9.0, 36.4; 0.0–51.5)	(8.6, 36.0; 0.3–87.0)	
Number (%) of patients undergoing sedation hold during the intervention period	30/36 (83)	33/38 (87)	0.751
Number (%) of patients extubated during the intervention period	17/36 (47.2 %)	17/38 (44.7 %)	1.00
Proportion of hours in intervention period during which patient extubated, median	4	0	0.49
(1st, 3rd quartile; min– max)	(0–62; 0–92)	(0, 44; 0–97)	
Number (%) of patients with any predefined adverse events during intervention period	7/36 (19.4 %)	5/38 (13.1 %)	0.54
Unplanned extubation, number	0	1	
Unplanned removal of vascular catheter, number	0	0	
Unplanned removal of nasogastric or other enteral tube, number	1	0	
Unplanned removal of other drain or device, number			
Episode of myocardial ischaemia, number	0	0	
Myocardial infarction, number	0	0	
Episode of agitation requiring bolus treatment with haloperidol or sedative drug (rescue medication), number	0	0	
	6	4	
Outcomes at 7 days post-randomisation			
Time from start of monitoring to first extubation, hours, median	42.4	54.8	0.52
(1st, 3rd quartile; min– max)	(14.7, 168.0; 2.0–168.0)	(22.8, 168.0; 1.78–168.0)	
Deaths, number (%)	1/36 (2.8 %)	3/38 (7.9 %)	0.62
Discharged from ICU, number (%)	20/36 (55.6 %)	18/38 (47.4 %)	0.50
Still in ICU, number (%)	15/36 (41.7 %)	15/38 (41.7)	1.00
Transferred to another ICU, number (%)	0/36 (0 %)	2/38 (2 %)	0.49

The intervention period was defined as being from the start of monitoring to 48 hours post ICU admission. *RI* Responsiveness Index, *RASS* Richmond agitation-sedation scale

**Table 3:** Comparison of outcomes for patients analysed in the post hoc exploratory analysis with baseline Responsiveness Index (RI) <20

Outcome	Intervention (n = 19)	Control (n = 20)	*P* value
Proportion of hours during the intervention period with RI value <20, median	16	51	0.02
(1st, 3rd quartile; min–max)	(11, 45; 2–87)	(33, 72; 5–86)	
RASS score at baseline, median	−4	−4	0.76
(1st, 3rd quartile; min-max)	(−4, −3; −5 to −2)	(−4, −3; −5 to 0)	
Proportion of hours with RASS score −4 or −5 during intervention period, median	47	35	0.63
(1st, 3rd quartile; min-max)	(15, 65; 0–94)	(10, 72; 0–100)	
Total propofol dose (mg) during intervention period, median	1,090	2,380	0.14
(1st, 3rd quartile; min-max)	(375, 2,965; 100–7,290)	(1,510, 3,730; 60–8,630)	
Total alfentanil dose (mg) during intervention period, median	21.2	32.3	0.01
(1st, 3rd quartile; min-max)	(8.5, 27.9; 0.0–49.0)	(23.3, 49.8; 1.0–87.0)	

*RASS* Richmond agitation-sedation scale

### Qualitative study

Nursing staff valued the objective visible representation of sedation state, the simple colour representation of trends, and the continuous prompt to manage sedation in contrast to existing intermittent assessments or interventions. The trending was seen as a useful alert and warning, for example, during sedation reduction/interruption. The objective data were also valued in justifying decision-making with medical staff, especially in relation to wakeful or agitated patients. Nurses valued the use of the monitor as an adjunct to decision-making rather being asked to target specific RI values. A full description of the qualitative data analysis and conclusions is provided in the additional material (see Additional file 2).

## Discussion

We report the first use of RI monitoring to support nurse decision-making in relation to early ICU sedation. Our data suggest that low RI values can safely be used as a prompt to decrease sedative drug use without increasing adverse events. The trend towards lower RI values during the intervention period with RI monitoring, which was statistically significant for the post hoc subgroup analysis of patients with baseline RI <20, suggest RI monitoring has potential utility for minimising unnecessary early deep sedation.

The RI index utilises fEMG data from the preceding 60 minutes to provide an overall measure of individual responsiveness [22]. Our validation studies found that this was less subject to the “on-off” fEMG artifacts that can confound the bispectral index and entropy, which are primarily based on EEG data [17, 21]. RI is intended to provide an objective integrated measure of patient arousal in relation to underlying neurological status, external stimulation, and administered sedation and analgesia over the preceding 60 minutes. RI values are low during deep sedation, but can also be low in other forms of coma and natural sleep, and during periods of minimal external stimulation [20]. These non-linear relationships with sedation and neurological status were the rationale for using low RI values as a prompt or alert, rather than using specific values as a sedation target. Despite the need to explore the potential reasons for a low RI value during decision-making, the interviewed nurses valued the information. The continuous objective simple presentation using colour in addition to numeric data was considered useful, and data were felt to support decision-making in relation to sedation reduction, but also to justify maintenance of the current sedation state. These qualitative findings further supported the RI concept, especially as many nurses had relatively limited experience using the technology when interviewed.

This was a proof-of-concept study, and a convenience sample size was used rather than a calculated sample size to demonstrate clinically important differences in patient outcomes. Non-significant trends towards quicker extubation, higher RI values and lower sedative drug use were observed in the intervention group, which mainly occurred in the subgroup in whom RI values were <20 at baseline, in whom there was greatest plausibility for an effect on clinical management. The frequent use (>80 %) of sedation holds and a protocol that included prompted hourly sedation assessment using the RASS score make it possible that the best practice provided in both groups decreased the effect size attributable to RI monitoring. However, our aim in this proof-of-concept study was to explore the safety and potential effectiveness of RI monitoring as an incremental intervention. These preliminary data are consistent with a potential utility for RI monitoring as an adjunct to regular clinical assessments, even when these are used frequently, but further studies are needed to evaluate the effectiveness of RI monitoring in larger and more varied ICU settings.

We did not observe significant differences in the RASS scores either for the overall cohort, or for the analysis restricted to patients with RI <20 at the start of monitoring. The relatively small patient numbers, wide variation in RASS scores, categoric nature of RASS data, and low overall proportion of time with RASS < −4 during the 48-hour intervention period may explain this finding. Our previous validation studies clearly indicate that clinical sedation scales, which score responses to specific stimuli at defined time points, are not directly comparable to the RI, which is a measure of overall arousal during clinical management over the previous 60 minutes of care [20, 21]. Although the RASS score has good discriminant ability and reliability in published studies [23], it is potentially subject to recording bias by clinical staff and is semi-subjective. Our proof-of-concept study provides quantitative and qualitative data suggesting that the objective RI data could influence nursing staff despite the frequent (hourly) recording of RASS score. However, further larger studies are needed to determine whether RI monitoring can increase overall RASS scores, and whether this translates into improved sedation quality and clinical outcomes.

Current evidence-based guidelines do not recommend the routine use of technologies to guide sedation management [13]. Published studies with existing technologies suggest variable discriminant validity, and problems with artefact suppression and confounding of algorithms by fEMG frequencies [16, 24, 25]. There are few published trials of technology-guided sedation and those published do not demonstrate benefit [19]. The RI was iteratively developed to address the problems encountered with other technologies, but further trials with power to detect differences in clinically important outcomes are needed to further evaluate its clinical effectiveness and cost-effectiveness.

Strengths of our trial include the prospective measurement of multiple proof-of-concept and safety outcomes, and the inclusion of a nested qualitative study with clinical users. The direct comparison of RI data between the groups with blinding of RI data from clinical staff in the control group reduced the chance of bias for this outcome. Weaknesses include the limited sample size, and the exclusion from analysis of randomised patients in whom subsequent consent could not be obtained. However, for a proof-of-concept trial in which comparisons of patients treated as per protocol was of the greatest interest, we think it unlikely that this was an important source of bias.

## Conclusions

In conclusion, we have shown that the use of RI monitoring as an adjunct to decision-making by bedside nurses has potential utility for improving sedation-management without increasing sedation-related adverse effects. The technology was acceptable and considered potentially useful by ICU nurses.

## Key messages

- The Responsiveness Index is a novel sedation-monitoring technology that may have clinical utility for modifying nurse decision-making in mechanically ventilated sedated critically ill patients
- Altering sedation using Responsiveness-Index-guided decision support did not increase sedation-related adverse events
- Nursing staff found Responsiveness Index monitoring a useful adjunct to decision-making, particularly the objective continuous data and simple colour-coded data presentation
- Larger trials of sedation management using Responsiveness Index monitoring are justified

## Additional files

Additional file 1:
**Pilot trial protocol for a randomised controlled trial of intensive care management of sedation using patient responsiveness in critical care (IMPROVE).** (PDF 399 kb) Additional file 2:
**Summary of qualitative analysis of interviews with ICU nurses regarding their views and opinions of responsiveness index monitoring in the IMPROVE pilot trial.** (PDF 288 kb)

## Abbreviations

APACHE: acute physiology and chronic health evaluation
CRF: case record file
EEG: electroencephalogram
fEMG: facial electromyelogram
ICU: Intensive Care Unit
NMBA: neuromuscular blocking agents
RASS: Richmond agitation-sedation scale
RI: Responsiveness Index
SOFA: sequential organ failure assessment

## References

[1] Shehabi Y, Bellomo R, Reade MC, Bailey M, Bass F, Howe B (2012). Early intensive care sedation predicts long-term mortality in ventilated critically ill patients. Am J Respir Crit Care Med.

[2] Watson PL, Shintani AK, Tyson R, Pandharipande PP, Pun BT, Ely EW (2008). Presence of electroencephalogram burst suppression in sedated, critically ill patients is associated with increased mortality. Crit Care Med.

[3] Strom T, Martinussen T, Toft P (2010). A protocol of no sedation for critically ill patients receiving mechanical ventilation: a randomised trial. Lancet.

[4] Kress JP, Pohlman AS, O'Connor MF, Hall JB (2000). Daily interruption of sedative infusions in critically ill patients undergoing mechanical ventilation. N Engl J Med.

[5] Girard TD, Kress JP, Fuchs BD, Thomason JW, Schweickert WD, Pun BT (2008). Efficacy and safety of a paired sedation and ventilator weaning protocol for mechanically ventilated patients in intensive care (Awakening and Breathing Controlled trial): a randomised controlled trial. Lancet.

[6] Brook AD, Ahrens TS, Schaiff R, Prentice D, Sherman G, Shannon W (1999). Effect of a nursing-implemented sedation protocol on the duration of mechanical ventilation. Crit Care Med.

[7] Hager DN, Dinglas VD, Subhas S, Rowden AM, Neufeld KJ, Bienvenu OJ (2013). Reducing deep sedation and delirium in acute lung injury patients: a quality improvement project. Crit Care Med.

[8] Quenot JP, Ladoire S, Devoucoux F, Doise JM, Cailliod R, Cunin N (2007). Effect of a nurse-implemented sedation protocol on the incidence of ventilator-associated pneumonia. Crit Care Med.

[9] Klompas M, Anderson D, Trick W, Babcock H, Kerlin MP, Li L (2015). The Preventability of Ventilator-associated Events. The CDC Prevention Epicenters Wake Up and Breathe Collaborative. Am J Respir Crit Care Med.

[10] Miller MA, Krein SL, George CT, Watson SR, Hyzy RC, Iwashyna TJ (2013). Diverse attitudes to and understandings of spontaneous awakening trials: results from a statewide quality improvement collaborative*. Crit Care Med.

[11] Gill KV, Voils SA, Chenault GA, Brophy GM (2012). Perceived versus actual sedation practices in adult intensive care unit patients receiving mechanical ventilation. Ann Pharmacother.

[12] Rose L, Fitzgerald E, Cook D, Kim S, Steinberg M, Devlin JW, et al. Clinician perspectives on protocols designed to minimize sedation. J Crit Care. 2014. doi:10.1016/j.jcrc.2014.10.021. 10.1016/j.jcrc.2014.10.021 25466317

[13] Barr J, Fraser GL, Puntillo K, Ely EW, Gelinas C, Dasta JF (2013). Clinical practice guidelines for the management of pain, agitation, and delirium in adult patients in the intensive care unit. Crit Care Med.

[14] Barr J, Pandharipande PP (2013). The pain, agitation, and delirium care bundle: synergistic benefits of implementing the 2013 Pain, Agitation, and Delirium Guidelines in an integrated and interdisciplinary fashion. Crit Care Med.

[15] Robinson BR, Berube M, Barr J, Riker R, Gelinas C (2013). Psychometric analysis of subjective sedation scales in critically ill adults. Crit Care Med.

[16] LeBlanc JM, Dasta JF, Kane-Gill SL (2006). Role of the bispectral index in sedation monitoring in the ICU. Ann Pharmacother.

[17] Walsh TS, Ramsay P, Lapinlampi TP, Sarkela MO, Viertio-Oja HE, Merilainen PT (2008). An assessment of the validity of spectral entropy as a measure of sedation state in mechanically ventilated critically ill patients. Intensive Care Med.

[18] Haenggi M, Ypparila-Wolters H, Bieri C, Steiner C, Takala J, Korhonen I (2008). Entropy and bispectral index for assessment of sedation, analgesia and the effects of unpleasant stimuli in critically ill patients: an observational study. Critical Care (London, England).

[19] Weatherburn C, Endacott R, Tynan P, Bailey M (2007). The impact of bispectral index monitoring on sedation administration in mechanically ventilated patients. Anaesth Intensive Care.

[20] Walsh TS, Everingham K, Frame F, Lapinlampi TP, Sarkela MO, Uutela K (2014). An evaluation of the validity and potential utility of facial electromyelogram Responsiveness Index for sedation monitoring in critically ill patients. J Crit Care.

[21] Walsh TS, Lapinlampi TP, Ramsay P, Sarkela MO, Uutela K, Viertio-Oja HE (2011). Responsiveness of the frontal EMG for monitoring the sedation state of critically ill patients. Br J Anaesth.

[22] Lapinlampi TP, Viertio-Oja HE, Helin M, Uutela KH, Sarkela MO, Vakkuri A (2014). Algorithm for Quantifying Frontal EMG Responsiveness for Sedation Monitoring. Can J Neurol Sci.

[23] Sessler CN, Gosnell MS, Grap MJ, Brophy GM, O'Neal PV, Keane KA (2002). The Richmond Agitation-Sedation Scale: validity and reliability in adult intensive care unit patients. Am J Respir Crit Care Med.

[24] Vivien B, Di Maria S, Ouattara A, Langeron O, Coriat P, Riou B (2003). Overestimation of Bispectral Index in sedated intensive care unit patients revealed by administration of muscle relaxant. Anesthesiology.

[25] Walsh TS, Ramsay P, Kinnunen R (2004). Monitoring sedation in the intensive care unit: can “black boxes” help us?. Intensive Care Med.

