# Supplementary material for: A randomized controlled proof-of-concept trial of early sedation management using Responsiveness Index monitoring in mechanically ventilated critically ill patients
Source: Crit Care. 2015 Sep 11;19(1):333. doi: 10.1186/s13054-015-1043-1 (PMC4570737; doi:10.1186/s13054-015-1043-1)
Supplement: Additional file 1: — Pilot trial protocol for a randomised controlled trial of intensive care management of sedation using patient responsiveness in critical care (IMPROVE). (PDF 399 kb) [file 13054_2015_1043_MOESM1_ESM.pdf]

A Randomised Controlled Trial of Intensive Care Management of Sedation using Patient Responsiveness in Critical Care

IMPROVE Critical Care study Pilot

Study Sponsor: GE Healthcare Finland Oy

Version 1.1
Date: 4th February 2010

Study Team

Chief Investigator:

Professor Tim Walsh
Royal Infirmary of Edinburgh
Little France Crescent
Edinburgh
Scotland
EH16 4SA
twalsh@staffmail.ed.ac.uk

Research Coordinator/PhD student

Ms Kirsty Everingham
Royal Infirmary of Edinburgh
Little France Crescent
Edinburgh
Scotland
EH16 4SA
kirsty.everingham@ed.ac.uk

GE Healthcare Team (Helsinki, Finland)

Petra Peltola, RN, Clinical Trial Manager, GE Healthcare

Hanna Viertiö-Oja, PhD (Eng.), GE Healthcare

Kimmo Uutela, PhD (Eng.), GE Healthcare

Petteri Lapinlampi, GE Healthcare

Lasse Kamppari, PhD (Eng.), GE Healthcare

Alan Davies, PhD MD, GE Healthcare

Daniel Jackson, GE Healthcare

Background

Overview

There is increasing evidence that sedation practice affects the outcome of critically ill patients. Specifically, over sedation is associated with prolonged mechanical ventilation and ICU stay, higher complication rates, and possibly excess mortality. At present, guidelines suggest that sedation should be managed using clinical scoring systems, linked to protocols that include titration of drugs and periods of sedation reduction or withdrawal. There are currently no sedation monitoring devices in widespread use in ICUs, and no device has been subjected to rigorous controlled trials.

GE Healthcare, in collaboration with the Edinburgh Critical Care Research Group, Scotland (UK), has developed a monitoring device based on responsiveness of the facial EMG. The device generates a responsiveness number based on facial EMG over the previous 60 minutes (weighted to most recent data). Completed studies suggest that the device is a valid measure of conscious level in response to ongoing treatment for sedated critically ill patients. This study protocol describes a prospective.unblinded randomised pilot trial comparing sedation management using a protocol based on responsiveness with standard sedation management. The hypothesis is that responsiveness will improve a range of patient-based and economic outcomes, including the duration of mechanical ventilation and duration of coma in the ICU.

Methods of monitoring sedation

Sedation scores

Clinical sedation scales use response to clinical stimuli to evaluate and record the sedation status of ICU patients. Most use a sequential series of stimuli starting with visual observation, progressing through response to voice, and subsequently mechanical and/or painful stimuli. The ideal sedation scale has validity, especially for discrete sedation states, is easy to administer, and importantly has high intra- and inter-rater reliability[1]. A large number of different sedation scales have been described, and subject to varying levels of assessment. Commonly used scales include the Ramsay scale [2], the Sedation Agitation Scale [3] the Motor Activity Assessment Scale [4], the Richmond Agitation-Sedation Scale (RASS) [5] the Adaptation to the Intensive Care Environment instrument (ATICE) [6], and the Minnesota Sedation Assessment Tool (MSAT) [7]. In addition, numerous modifications of these scales or locally developed tools are in use in ICUs. There is no universally agreed sedation assessment tool either nationally or internationally. However, the most widely validated tool, with publications showing very high intra and inter-rater reliability, is the RASS score [8].

Existing Processed EEG parameters

Bispectral index

The Bispectral Index (BIS) was developed as a depth of anaesthesia monitor. A large number of publications have examined its clinical value in this setting [9]. Two large trials specifically evaluated whether BIS could decrease the incidence of patient awareness under anaesthesia. One trial indicated a reduction in awareness, although a more recent trial found no difference with a control group [10, 11]. A number of studies have evaluated the use of BIS in the ICU setting [12]. Although several of these studies show apparent linear correlations between BIS numbers and clinical sedation categories a common finding is a wide range of values within each sedation state, indicating poor discrimination. Specifically, high BIS numbers are frequently seen in unresponsive patients, suggesting low specificity for deep sedation. Some studies suggest this is due to EMG artifact, even with newer versions such as BIS-XP [13], [14]. In some studies the administration of neuromuscular blockade without changing sedative drug infusions resulted in large decreases in BIS numbers [15]. One recent randomised trial in 50 patients found that a sedation protocol base on BIS monitoring and values actually increased sedative use over time compared to standard practice [16]. Recent surveys suggest that BIS is rarely used for routine sedation monitoring in ICUs [17].

Entropy

Spectral entropy was developed by GE Healthcare as a means of monitoring depth of anaesthesia and has been licensed for this clinical use. A range of publications indicate its utility as a measure of depth of anaesthesia and favourable comparisons with BIS monitoring. Entropy was investigated through the collaboration between GE Healthcare and Edinburgh University as a potential monitor for depth of sedation in the ICU. These studies concluded that entropy had poor discrimination between different clinical sedation states, and between deeper and lighter sedation states[18]. The main confounder was found to be facial EMG activation, which was highly prevalent in ICU patients and correlated closely with clinical sedation state. Entropy has not been promoted as a sedation monitor for ICU patients.

Responsiveness

Rationale for responsiveness

Responsiveness has been developed from first principles through a collaborative research programme between GE Healthcare and the Edinburgh Critical Care Research Group. Development has been based on detailed annotated data files of raw EEG/EMG data acquired from forehead electrodes in ICU patients in whom clinical sedation status was recorded by a single trained observer. Early studies clearly showed that EMG was a major confounder to EEG based algorithms. Existing “black box” devices were adapted or transferred from the anaesthesia setting [19]. Consideration of the aims of sedation monitoring in anaesthesia and ICU indicates that the goals are different, and show why EMG artifact is likely to be problematic with existing devices (Table 1).

Goal for Anaesthesia monitoring	Goal for ICU monitoring
• Goal is deep anaesthesia with non-responsiveness<br>• Facial EMG activity useful as an index of arousal or "light anaesthesia"<br>• The goal is detection and avoidance of undersedation/hypnosis<br>• Limited range of nociceptive stimuli<br>• Movement rare	• Goal is light sedation with responsiveness<br>• Facial EMG problematic as optimum sedation infers active fEMG<br>• The goal is avoidance of oversedation/hypnosis<br>• Frequent and varied nociceptive stimuli<br>• Movement common

Table 1. Goals for Anaesthesia vs ICU monitoring

These factors led to a fundamental re-evaluation of the approach to monitoring. It was acknowledged that facial EMG responsiveness during ICU care results from the complex interaction of several factors:
[1] the intensity and frequency of stimulations of the patient, for example from physiotherapy, mechanical ventilation, or the underlying condition (including pain).
[2] the sedative and analgesic drugs received by the patient (particularly the brain concentrations actually present, which may be difficult to predict due to altered pharmacokinetics and dynamics).
[3] the effect of the illness and drug treatment on brain function, specifically the presence of encephalopathy and/or delirium, which effects up to 80% of ICU patients and may alter responses of patients to stimulation, and the performance of clinical sedation scales.

A hypothesis was generated that the responsiveness of the facial EMG would be a useful dynamic and continuous measure of the balance between sedation requirements and actual sedation state, where:

- "high responsiveness" indicated either the awake state or an undersedated state in a patient receiving high levels of stimulation
- "low responsiveness" indicated either the asleep state or coma due to the underlying condition or excessive sedation in relation to the level of stimulation

A useful sedation monitor requires good discrimination between low and high responsiveness with high specificity that low responsiveness indicates the clinical need to decrease sedation dose in patients receiving sedative drugs.

The responsiveness algorithm has been developed through an iterative process to maximise discrimination between different clinical sedation states and to maximise face validity against detailed clinical data files. Artifact suppression algorithms have been included. In addition, sensitivity analysis has been used to produce best cut-off values for deeper sedation states, intermediate states, and awake states. These have been converted to a

“traffic light” system whereby red equates to low, amber to intermediate, and green to awake/easily rousable levels of responsiveness.

The responsiveness monitoring device that will be used in this study will be CE marked.

Existing trials of sedation management in intensive care

Sedation protocols

A summary of published studies examining sedation practice is shown in appendix 1.

Five RCTs have compared a sedation protocol with a control practice.

Brook and colleagues (1999) compared physician orders with a nurse led protocol in a single US medical ICU. Weaning practice was not standardised and there was no use of sedation breaks. The authors found a statistically and clinically important decrease in duration of ventilation, ICU and hospital stay and a reduction in tracheostomy use.

Kress and colleagues (2000) compared standard practice, which did not include sedation scoring or pre-defined protocols, with a daily sedation break in medical ICU patients in a single US ICU. The authors found a significant decrease in duration of ventilation and ICU stay but not hospital discharge. There was no excess of adverse events in the intervention group and a significant reduction in investigations for persistent coma. Subsequent follow up studies found a trend to lower incidence of long term post-traumatic stress disorder (PTSD) symptoms and better adaptation to illness in the group in whom daily awakening was carried out [20]. There was no standardisation of weaning practice. In another retrospective analysis of this data base [21] the authors examined the incidence of ventilator associated pneumonia, upper GI haemorrhage, bacteraemia, barotrauma, venous thromboembolism, cholestasis, and sinusitis and found lower cumulative incidence in the daily sedation hold group (2.8 versus 6.2%). A related observational study suggested that although ischaemic myocardial events occur in 24% of medical patients in ICU this was not increased during daily sedation breaks [22].

Carson and colleagues[23] randomised patients in two medical ICUs to receive intermittent lorazepam and daily sedation hold or continuous propofol infusion plus daily sedation hold. Although both groups received daily sedation holds on 82% of days the duration of mechanical ventilation was significantly shorted in the propofol group, and for survivors ICU stay was shorter. Patients in the lorazepam group were more sedated during weaning trials and had a higher rapid shallow breathing index values consistent with lower probability of weaning from the ventilator.

Recently Girard and colleagues[24] compared standard practice, which did not include daily sedation breaks, with a protocol combining daily sedation holds and weaning trials in four medical ICUs in the US. The control group practice included clinical sedation scoring and individual unit protocols. The authors found a significant decrease in duration of ventilation, ICU, and hospital stay. They also observed a statistically and clinically important decrease in mortality at 1 year post-randomisation, although the survival curves separated after the period of intervention. An important difference between the groups was significantly greater sedation in the control group at the time of first weaning trial, and shorter duration of coma in the intervention group.

A recently published single centre Australian trial comparing non-protocolised with protocolised sedation practice found no difference in outcomes between groups [25]. One argument proposed for this negative finding was differences in staffing experience and intensity of work compared to other health care systems.

A number of before and after studies and practice improvement initiatives have shown a range in clinical effects from implementing sedation protocols with varying designs (see appendix 1)

Current sedation practice in intensive care units

Recent surveys of practice

Several recent surveys have investigated sedation practices in ICUs. These include surveys of clinicians' stated views, and actual measurement of practice. A survey of 904, mainly US, members of the Society of Critical Care Medicine published in 2004, which included physicians, nurses and pharmacists, found that 40% of these professionals stated they used daily sedation interruption, 62% used a sedation scale, and 64% stated they had sedation protocols established in their ICUs [26]. A German survey of 261 hospitals, published in 2005, found that a written standard operating procedure (analogous to a protocol) for sedation and analgesia existing in only 21% of hospitals; although 30% of hospitals stated they monitored sedation only 8% identified a specific scale for sedation [27] Older surveys in Denmark and Sweden carried out > 5 years ago found that sedation protocols were used in <30% of patients and specific scoring systems in <20% of patients [28], [29]. A survey of Canadian critical care practitioners was published in 2006, but undertaken in 2001 [17]. This showed that only 40% of practitioners practiced daily interruption of sedation, only 49% used a sedation scoring system, and 29% used a protocol to guide sedation practices in the ICU. The frequency of sedation scoring varied from hourly (29%), to 4 hourly (41%), once per shift (10%), and 15% stated frequency depended on individual clinicians or patients. The most recent survey of practice was carried out in 44 French ICUs over 12 months from January 2004 to January 2005 [30]). This study used direct observation of practice in patients on days 2, 4, and 6 of ICU stay. The observed rates of sedation assessment were only 43%, 36%, and 31% on days 2, 4, and 6 respectively despite 72%, 56%, and 49% of patients receiving sedative drugs on these days, indicating that 30-40% of

patients received sedation with no assessment. Only 16 of 44 sites had a sedation/pain management guideline/protocol in place and no sites were conducting daily interruption of sedation.

Together these surveys of clinicians and clinical practice indicate wide variability in the use of sedation scales, protocols and daily sedation breaks. There also appears to be wide variation in the frequency of sedation scoring systems, the type of system used, and the composition of sedation protocols.

Published recommendations regarding sedation practice

For this randomised trial, a key issue is the practice that is used to guide sedation practice in the control group. The control group is intended to be consistent with current recommended best practice, but also to reflect commonly used sedation practice. Recent surveys of sedation practice (see above) show a wide variation in current practices, which raises the possibility of discordance with published best practice guidelines. This has been recognised as a common problem in critical care, where translation of evidence into routine practice has been poor. As the aim of responsiveness monitoring is to improve sedation practice, it is essential that the control group is [a] a reasonable reflection of best practice principles, and [b] that the actual control group practice is described in detail in relation to the key elements of best practice.

We reviewed the literature relating to the following key questions and drew conclusions that were incorporated in to the minimum requirements for the control group practice.

What was control group practice in the key published RCTs of sedation practice in ICUs?

In the Brook study[31] the control group sedation practice was based on physician orders, and there was no evidence of use of clinical sedation scales, a protocol, or sedation breaks. In the Kress study[21] the control group received sedation as dictated by the clinical team. There was no evidence of clinical sedation scoring or protocols, and the intervention group received the sedation breaks. In the Girard study[24] the control group were managed with individual unit protocols with clinically targeted sedation states, but it is stated that clinical sedation assessment was undertaken routinely. Sedation breaks were only undertaken in the intervention group.

We conclude from these studies, which all showed clinically and statistically important treatment effects in the intervention group, that a control group practice which includes clinical sedation scoring, sedation protocols, and daily sedation breaks has not been used in previous RCTs.

What do published studies and guidelines state regarding the frequency of clinical sedation scoring in ICU patients?

We reviewed a large range of clinical studies and guidelines that considered clinical sedation scoring. These are summarised in appendix 2. There was no consensus as to how frequently clinical sedation scoring should be

undertaken. There is no evidence from trials indicating how frequently clinical sedation scoring should be undertaken.

What do published studies and guidelines state regarding daily sedation breaks?
Two studies with major treatment effects, the Kress study[21] and Girard study[24], included daily sedation holds in the intervention arm. US guidelines strongly recommend the use of daily sedation breaks [32]. The most recently published evidence based guidelines are the surviving sepsis guideline [33]. This includes the following statements and grades of evidence:

Table 2: recommendations relating to sedation management from the 2008 surviving sepsis campaign

	Grade of recommendation
We recommend sedation protocols with a sedation goal when sedation of mechanically ventilated patients with sepsis is required	1B
We recommend intermittent bolus sedation or continuous infusion sedation to predetermined end points (e.g. sedation scales) with daily interruption/lightening of continuous infusion sedation with awakening and retitration if necessary for sedation administration to septic mechanically ventilated patients	1B

Grade 1 = strong recommendation
Grade B = downgraded RCT or upgraded observational study

Together, this literature indicates that for a trial control group to reflect "best practice" the management should include:
[1] The use of a clinical sedation scale, but the exact scale used and the frequency of measurement do not need to be pre-defined or controlled
[2] The presence of a protocol in each ICU that links clinical sedation scores to decision-making, although tight control over the use of the protocol is not required to reflect current actual clinical practice.
[3] Consideration of daily sedation interruption or lightening for patients receiving continuous sedation, and re-titration of drug doses.

Existing evidence does not support routine use of any existing sedation monitoring device.

Summary of existing literature

- Published trials indicate that a systematic organisational change can produce clinically important reductions in duration of mechanical ventilation, ICU and hospital stay.
- Avoiding oversedation in association with consistent approaches to weaning should be central goals of intensive care management

- Using valid clinical sedation scales, preferably linked to sedation protocols can improve patient outcomes
- Daily sedation holds (“daily wake up”) is one clinically effective method of avoiding excessive sedation use
- Expert opinion and guidelines recommend the use of clinical sedation scores linked to sedation protocols, and the use of daily sedation breaks as “best practice”
- The use of daily wake up, clinical sedation scores, and sedation protocols remains highly variable in clinical practice
- No existing medical device for sedation monitoring has been shown to improve clinical outcomes in ICU patients

The need for a trial
The previous literature review indicates the importance of optimising sedation in critically ill patients generally and avoiding over-sedation specifically. Data also suggest that although best practice guidelines exist, compliance with these is poor worldwide. Health technology that could improve compliance with best practice, or offer an alternative approach to detect over-sedation and prompt staff to decrease sedative drugs could be clinically effective. EMG responsiveness is a novel health technology designed to detect low patient responsiveness during ICU treatment. There is a clear need to evaluate the clinical and cost-effectiveness of this technology in practice. Specifically, a trial needs to evaluate whether the device increases health care worker confidence; increases therapeutic decision making in relation to sedation, and improves patient outcomes.

The Trial

Design and methodology
This is a pilot unblinded randomised controlled trial comparing sedation management using responsiveness (new intervention) with usual care (control group).

Study objectives

Pilot study
1. To test the protocol in the clinical setting
2. To determine recruitment rates to the study
3. To assess adverse event rates in the intervention group compared with usual practice
4. To undertake a qualitative evaluation of the acceptability of the intervention protocol

Full study
<u>Primary objective</u>
To evaluate whether decision-making based on responsiveness can decrease the proportion of time patients spend with low responsiveness values during the first 48 hours of intensive care management.

Secondary objectives

1. To investigate whether monitoring the sedation state of mechanically ventilated critically ill patients with the responsiveness monitor *reduces the duration of coma* compared with usual sedation practice.
2. To investigate whether monitoring the sedation state of mechanically ventilated critically ill patients with the responsiveness monitor *reduces the duration of mechanical ventilation* compared with usual sedation practice
3. To investigate whether monitoring the sedation state of mechanically ventilated critically ill patients with the responsiveness monitor is *associated with excess adverse* events compared with usual sedation.
4. To undertake a *qualitative evaluation of nurse decision-making* in relation to responsiveness monitoring

Trial design

Prospective single-centre randomised unblinded controlled pilot trial

Hypothesis

Our primary hypothesis is that continuous monitoring of EEG/EMG responsiveness linked to continuous bedside prompting via a decision-making algorithm can decrease the time patients spent in the lowest levels of responsiveness ("red zone") during the first 48 hours of ICU management (or when first ventilated and sedated in ICU) compared to a control group that represents current best practice.

Eligibility/Entry Criteria

Inclusion criteria

[1] Patient mechanically ventilated via an endotracheal tube
[2] Patient receiving intravenous sedation with a hypnotic agent (midazolam or other benzodiazepine) or propofol by continuous infusion.

Exclusion criteria:

1. Primary intracerebral disorder (includes cardiac arrest with probable hypoxic brain injury; intracranial haemorrhage; head injury causing reduced conscious level prior to intubation)
2. Age <16 depending on local guidelines and ethical committees
3. Patient not expected to survive the next 24 hours
4. Patient receiving long term ventilation prior to ICU admission
5. Patient with a long term tracheostomy prior to ICU admission
6. Patient transferred sedated and mechanically ventilated from another ICU unless recruitment is possible within 12 hours of first ICU admission
7. Patient receiving continuous neuromuscular blocking agent at the time of screening for enrolment.
8. Previously enrolled in the trial during a separate ICU admission during this hospital stay.
9. Status epilepticus

10. Confirmed meningitis or encephalitis at the time of screening for enrolment
11. Chronic neurological disease (excluding previous CVAs) interfering with normal neuromuscular function, e.g. motor neurone disease, Guillain-Barre syndrome or inherited neuromyopathies
12. Patient who has been ventilated and sedated > 12hrs in ICU at time of screening
13. Patient who is being weaned from the ventilator with the aim to extubate in the next 12 hours, as per Dr instructions

Patient recruitment and flowchart

All sequential admissions to the ICU during the study period will be screened at the time of admission. We will aim to enter patients to the trial from the time of ICU admission, seeking delayed consent from relatives.

Ethical Issues

This study will by definition be undertaken with incapacitated patients. In addition, sedation is routinely started at the time of mechanical ventilation, which is usually the time of ICU admission. In order to have the greatest chance of testing the hypothesis the randomisation and implementation of the two strategies is needed at the time of initiating sedation. Our previous studies have shown that telephone consent prior to implementing sedation studies results in a mean delay of 14 hours (1st, 3rd quartile 10-12 hours) from ICU admission to implementing monitoring. We therefore require deferred consent to test the hypothesis, particularly as this study focuses on a fixed 48 hours time window. Clinical logic and experience also justifies immediate randomisation because sedation doses are often highest during the first 24 hours in the ICU. Improving practice in this period could decrease duration of coma and ventilation. Deferred consent from relatives/welfare guardians will be sought at the earliest opportunity. As the intervention will by definition have been completed at the time the patient regains capacity we do not propose to obtain patient consent.

Consent

Pending research ethics committee ruling, we will randomise patients at the time of mechanical ventilation and sedation with consent deferred for the reasons laid out above. In this respect this research is considered emergency research. We will approach relatives/welfare guardians for consent to remain in the study at the earliest opportunity. This will maximise recruitment (especially given the high proportion of patients admitted outside normal working hours) and give the greatest chance of testing the hypothesis.

Randomisation

Patients will be randomised by opening opaque sealed envelopes. All randomised patients will be included in the intention-to-treat analysis. We do not plan to stratify or minimise patients at randomisation. Block randomisation is not proposed for this single centre pilot study.

Management during intervention period

Intervention group: continuous responsiveness monitoring

Use of responsiveness monitor

The responsiveness monitor will be attached to the patient and data presented to the clinical staff continuously. All nursing staff caring for patients in the study will receive training in the study protocol and the use of the monitor prior to commencing the trial. A log will be kept of all staff caring for patients in the study in the case record file based on nursing shifts. This will ensure an audit trail that all staff had received training in monitor use, and relevant GCP to the trial.

The monitor will:
[a] Present a continuous trend over time that will be colour coded using a traffic light system.
[b] A responsiveness number will also be recorded representing the most recent responsiveness value, colour coded appropriately, in a separate window.
[c] An information/instruction box will be presented continuously to staff based on the current responsiveness value.

Table 3: Messages on the monitor screen.

Monitor colour	**Monitor instruction on screen**
Green	No coloured box<br><br>Text<br>Ensure adequate analgesia<br>Continue current sedation unless patient agitated
Amber	In coloured box<br>Moderate risk of oversedation<br><br>Text below box<br>Ensure adequate analgesia<br>Reduce sedation if no eye-opening to physical stimuli
Red	In coloured box<br>High risk of oversedation<br><br>Text below box<br>Ensure adequate analgesia if responsive to stimuli, eg suctioning/physiotherapy<br>Reduce sedation dose if no eye-opening to physical stimuli

Recording of sedation scores
The bedside nurse will perform a sedation assessment following a sequence of tests including standardised observations and stimuli that are listed on a paper form (see figure 1 for details); this is routine practice in ICU. This procedure generates a RASS score. The bedside nurse will enter the result on to the nurse log including the date and time of the assessment and his/her signature. In addition to the RASS score the nurse will be asked to record the responsiveness number, the colour of the responsiveness number on the monitor and whether they agreed or disagreed with the prompt issued by the monitor at this time. The nurse is asked to provide details of the instances why they disagree with the monitor suggestion. The RASS scores will be used to define the duration of clinical coma, which is one of the outcome measures.

No instruction regarding sedation changes will be linked to this scoring. However, nurses will be free to modify sedation dosing based on this or other forms of routine assessment at any time, as part of usual practice.

All clinical sedation scores performed by clinical staff during the intervention period are to be recorded on the nurse logs issued. These data will enable the frequency of recording to be described for each patient and enable comparison between intervention and control groups.

Figure 1: The RASS scoring system [8].

Table 1. The Richmond Agitation-Sedation Scale (RASS)

Score	Term	Description	
+4	Combative	Overtly combative, violent, immediate danger to staff	
+3	Very agitated	Pulls or removes tube(s) or catheter(s); aggressive	
+2	Agitated	Frequent nonpurposeful movement, fights ventilator	
+1	Restless	Anxious but movements not aggressive or vigorous	
0	Alert and calm		
−1	Drowsy	Not fully alert, but has sustained awakening (eye opening/eye contact) to voice (>10 seconds)	Verbal stimulation
−2	Light sedation	Briefly awakens with eye contact to voice (<10 seconds)	Verbal stimulation
−3	Moderate sedation	Movement or eye opening to voice (but no eye contact)	Verbal stimulation
−4	Deep sedation	No response to voice, but movement or eye opening to physical stimulation	Physical stimulation
−5	Unarousable	No response to voice or physical stimulation	Physical stimulation

Procedure for RASS Assessment

1. Observe patient
   - Patient is alert, restless, or agitated. — Score 0 to +4
2. If not alert, state patient's name and say to open eyes and look at speaker.
   - Patient awakens with sustained eye opening and eye contact. — Score −1
   - Patient awakens with eye opening and eye contact, but not sustained. — Score −2
   - Patient has any movement in response to voice but no eye contact. — Score −3
3. When no response to verbal stimulation, physically stimulate patient by shaking shoulder and/or rubbing sternum.
   - Patient has any movement to physical stimulation. — Score −4
   - Patient has no response to any stimulation. — Score −5

Adapted with permission.[29]

Conditions to disconnect or reattach responsiveness monitor
Responsiveness monitoring will be performed continuously until one of a pre-defined set of criteria to discontinue monitoring is met.

Criteria to discontinue responsiveness monitoring
Monitoring will be discontinued when one of the following occurs:

1. 48 hours have elapsed from ICU admission or from when first ventilated and sedated in ICU.
2. The patient is extubated.
3. The patient dies or a decision is made to withdraw treatment. In these cases the computer and monitoring equipment should be removed and an end-of-study entry made in the CRF.

Criteria to re-start responsiveness monitoring
If responsiveness monitoring has been discontinued, it should be re-attached and re-started if the patient requires re-intubation and sedation within the 48 hours intervention period

The goal will be to use responsiveness whenever the patient is intubated and intravenous sedation is administered. Responsiveness monitoring will continue during temporary sedation stops that are part of the care procedure.

Duration of assessing RASS score
Hourly RASS scoring will continue throughout the 48 hours period unless:

1. The patient dies or a decision to withdraw treatment is made
2. The patient is discharged from ICU (In these cases RASS will be assumed to be 0 or greater confirmed by patient visits)

Duration of intervention
The intervention will continue for up to 48 hours.
One of the following will be recorded as the reason for completing the intervention:

1. Patient is successfully extubated (monitoring will be re-instituted for patients re-intubated during 48 hours from first randomisation)
2. Patient dies in ICU prior to extubation within 48 hours
3. Patient having active treatment withdrawn prior to extubation within 48 hours
4. Patient transferred to another ICU before extubation within 48 hours
5. Relative consent declined

Control group: Usual practice including daily sedation group

Minimum standards for control patients for centres participating in the trial
There are no universally accepted standards for best sedation practice for ICUs. Based on review of current recommendations from national societies and guidelines (see earlier), current best practice for the control group will be defined as:

1. Use of a clinical sedation scoring system
2. A sedation protocol that links a clinical scoring system to suggested sedation management
3. Consideration of daily sedation holds/breaks

The frequency of performing and recording clinical sedation scores for the control group and intervention groups will not be defined *a priori*, but will be determined by local practice. It will not be modified for the purpose of the study. Actual practice will be recorded in the case record file on a daily basis.

Data capture in the control group
Data for responsiveness will be captured in a manner identical to the intervention group including the responsiveness monitor. However, all data will be blinded to the study staff and no instruction boxes will appear. A bedside computer will capture responsiveness data for all patients to enable comparison with the intervention group. Prompts to check electrode contacts will occur as in the intervention group, if a sensor check is necessary. Criteria to remove or re-attach the monitor will be identical to the intervention group. A nurse log will be completed in this group too, as in the intervention group, but will only gather date, time, RASS score given by nurse, any comments they have and their signature.

Daily data recorded in the CRF will be identical for both intervention and control groups.

Special situations

Surgery
Any patient requiring a surgical procedure either in the operating theatre or in the ICU will be managed using standard local practices for anaesthesia. Responsiveness monitoring will be removed from patients leaving the ICU for surgical procedures or other procedures or investigations (e.g. CT scanning, angiography) and re-instituted on return. The reasons for interruption of monitoring during clinical management will be recorded in the CRF, including a start and stop time.

Procedural sedation
Patients requiring procedures such as tracheostomy or drain insertion will be managed using standard local practices in both groups during the procedure. These interventions will be recorded in the CRF, including a start and stop time.

Patients receiving neuromuscular blocking drugs for the purpose of paralysis
Responsiveness is not valid for paralysed patients. Therefore when a patient requires paralysis usual practices for managing sedation will be employed. Patients will not be entered into the trial if they require paralysis at the time of enrolment. However, patients not paralysed at study enrolment who subsequently require a period of paralysis will continue in the trial and be analysed on intention to treat. During the period of paralysis usual sedation

practices will apply in both groups. Paralysis will be recorded in the CRF, including start and stop times.

Steps to minimise the effect of bias

Method of randomisation
Patients will be randomised on a 1:1 basis to the intervention or control group. No stratification or minimisation for other variables is proposed for the pilot study. Randomisation will be undertaken at the study site using sealed opaque envelopes.

Screening logs
A detailed enrolment log will be kept in the ICU of all sequential ICU admissions during the recruitment period. Reasons for non-eligibility will be recorded against the inclusion and exclusion criteria.

Control group practice
There will be no control of care in the control group, which is intended to reflect “usual care” in the individual centre. It is possible that practice could be altered either as a result of the study (for example better staff education or greater clinical interest in sedation practice) or over time as a result of experience gained during the trial. It is difficult to eliminate this possibility, but in order to explore potential bias due to a “study effect” in the control group detailed information regarding frequency of sedation scoring and daily sedation breaks will be recorded, together with daily clinical decisions relating to weaning.

Responsiveness monitoring will also be undertaken in the control group, but no data will be available to staff at the site. The randomisation group will ensure that data cannot be accessed during the intervention period for control patients. Availability of responsiveness for both groups will enable a fuller assessment of the effect of the technology on decision-making, specifically by comparing the duration of periods of low responsiveness (red code).

Recording coma outcomes
RASS scores obtained from clinical assessments will be used to compare the duration of coma between the groups. It is possible that this approach could introduce bias by prompting sedation assessment at times other than “standard practice” and that inter-rater variability could occur. However, inter-rater reliability has been shown to be extremely high for RASS scores. Although ideally all sedation assessments would be done by an independent blinded researcher this is not feasible, and could also introduce investigator bias because blinding from group allocation would be difficult. With the proposed approach group allocation of the coma measures will remain blinded from the statistician analysing the data.

Adverse events

This is a pilot study, and adverse event incidence is one of the outcomes. This is a trial of a medical device and as such is not subject to the guidelines set down in the European Clinical Trials Directive. Adverse event reporting will follow guidelines from the National Research Ethics Service for safety reporting in research other than clinical trials of investigational medicinal products.

Definition of a serious adverse event (SAE)
A SAE is an untoward and unexpected occurrence that a research participant experiences which:

1. Results in death
2. Is life threatening
3. Requires hospitalisation or results in prolongation of existing hospitalisation[1]
4. Results in persistent or significant disability or incapacity
5. Consists of a congenital anomaly or birth defect[2]

Note: [1] All patients in this trial will be hospitalised; [2]This is not relevant to this study population. In addition, all participants will, by enrolment criteria definitions, have life threatening illnesses that can result in death or persisting disability/incapacity.

Reporting of SAEs

Death is an expected serious adverse event during critical care treatment. As the intervention in this trial lasts for a maximum of 48 hours from randomisation it is not anticipated that deaths beyond 7 days post randomisation relate to the intervention. We will therefore only report deaths occurring within 7 days of randomisation as an SAE.

An SAE must be reported on the appropriate trial form by the clinician or research staff involved with the patient and reported locally immediately to the Principal Investigator at the centre. The PI at the centre must report the SAE to the Chief Investigator within 3 working days of the event. For the pilot study the PI is also the CI for the trial.

The Chief Investigator will give an opinion as to whether the event is [a] "related" (resulting from the administration of any of the research procedures), and [b] "unexpected" (the type of event is not listed in the protocol as an expected occurrence)

Any confirmed, related SAE will be reported to GE healthcare (the sponsor) within 24hrs of the research team becoming aware of it and it will be submitted to the relevant main Research Ethics Committee within 15 days of the Chief Investigator becoming aware of the event using the NRES report of serious adverse event form.

Expected adverse events

The patients enrolled in this study will already have a life threatening condition and judgement of SAEs and AEs is potentially problematic. Several adverse events could differ between the study groups. These have been predefined based on the study population, the nature of the intervention, and experience from previous trials of sedation in the ICU.

The following events will be considered expected adverse events in the study population and will be monitored prospectively:

1. Unplanned extubation
2. Unplanned extubation requiring re-intubation within 48 hours
3. Unplanned removal of vascular catheter
4. Unplanned removal of nasogastric/enteral tube
5. Episode of myocardial ischaemia
6. Myocardial infarction
7. Episode of agitation requiring pharmacologic treatment with sedative drug or haloperidol

These will be recorded systematically on a daily basis during ICU stay.

Outcome measures

Primary outcome measures

1. Recruitment rate (proportion of eligible patients)
2. Proportion of time spent with low responsiveness (red colour code) during the first 48 hours in ICU (or from first sedated and ventilated)
3. Proportion of time spent with RASS score -4/-5 during first 48 hours (or from when first sedated and ventilated).
4. Expected adverse event rates (comparison of control and intervention groups during intervention period)

Secondary outcome measures

1. Duration of mechanical ventilation
2. ICU, hospital mortality
3. Total sedative drug dose during first 48 hours in the ICU (or from when first sedated and ventilated)
4. Total dose of sedative drug during ICU stay (up to 7 days follow up)
5. Mean sedation drug use per day of mechanical ventilation (up to 7 days follow up)
6. Total opioid drug dose during first 48 hours in the ICU (or from when first sedated and ventilated)
7. Total opioid drug dose during ICU stay (up to 7 days follow up)
8. Mean opioid drug use per day of mechanical ventilation (up to 7 days follow up)

Sample size and duration of study

This is a pilot study so no formal sample size estimation has been performed. The Royal Infirmary of Edinburgh ICU admits 700 level (ventilated) patients each year. From local data we estimate that 500 patients will be potentially eligible for inclusion (9-10 per week). Assuming ethical approval to enrol patients at the time of ICU admission, with deferred consent from welfare guardians/relatives, and 20-30% enrolment of eligible patients we anticipate recruitment of 2-3 patients per week. We propose an 8 months recruitment period, recruiting a cohort of 60-70 patients (30-35 per group). We believe this will be sufficient to test the existing protocol, inform modifications, and provide data sufficient to power a full evaluation using a measure of coma as primary outcome.

Our aim will be to proceed to full trial, potentially on multiple sites, using data from the pilot study to inform modifications to the protocol.

Data collection

Baseline data
The following data will be entered into the CRF at the time of enrolment:
Age, gender
Main admission diagnosis to ICU
Date of hospital admission, date of ICU admission, time of ICU admission
APACHE II score for first 24 hours in ICU
Comorbidity scores: Charlson comorbidity index; functional comorbidity index (extracted from patient records)
Worst SOFA score for first 24 hours in the ICU
RASS score at time of randomisation
Sedation drug use in ICU prior to randomisation
Opiate drug use prior to randomisation

Electronic prompts
Internal sensor checks will be made every 10 minutes to check signal quality
Other electronic prompts will be displayed when signal is disrupted or is poor i.e. cables disconnect. A Frequently Asked Questions (FAQs) form has been devised displaying error messages and appropriate action that should be taken to rectify these.

Daily data recorded to CRF
The daily data collection during the intervention period is summarised below. This will be based on a 08:00am to 07:59 period each day and be entered each morning by research staff. There will be a part 24 hours period for the first period following randomisation, for which the duration will depend on the timing of randomisation. Daily data forms will be continued during ICU stay for up to seven days post randomisation.

Daily data collection on each day in ICU

Table 4. Collected daily data.

Data during past 24 hours (partial 24 hours for first)
Total sedation dose<br>Propofol<br>Midazolam<br>Other (define from pre-study survey)
Total Opiate dose<br>Morphine<br>Alfentanyl<br>Other (define from pre-study survey)
Other sedative drugs<br>Clonidine<br>Haloperidol
Inotropic/vasopressor drugs
Mode of ventilation<br>PaO2 at 8am (or closest sample)<br>FiO2 at time of 8am blood gas (or closest sample)
Weaning trials (for patients receiving mechanical ventilation)<br>Did the patient undergo a weaning trial during this period?
Did any of the following occur during the past 24 hours<br>Planned extubation (time)<br>Unplanned extubation (time)<br>Reintubation (time)<br>Unplanned removal of vascular catheter<br>Unplanned removal of nasogastric or other enteral tube<br>Unplanned removal of other drain or device (enter detail)<br><br>Episode of myocardial ischaemia<br>Myocardial infarction<br><br>Episode of agitation requiring bolus treatment with haloperidol or sedative drug ”rescue medication”<br><br>New infection (site)
Did patient receiving the following during previous 24 hours?<br>Tracheostomy
Procedures undertaken (use codes)

Weaning:<br>Has the patient had a sedation hold today?<br>Is the patient ready to wean today?<br>What is the highest mode of ventilation received today?	

Clinical sedation status during 24 hours period
All clinical sedation scores entered in the patient record will be transcribed into the daily CRF form.

Analysis

Primary outcome

1. Recruitment rate will be reported as a proportion of eligible patients. The reasons for non-recruitment of eligible patients will be reported
2. We will compare continuous responsiveness data during the first 48 hours using survival analysis. The proportion of the first 48 hours in the ICU (or when first sedated and ventilated) spent by patients in the “red” (unresponsive) zone will be compared between the groups using Mann Whitney tests
3. We will compare hourly RASS scores between the groups over the first 48 hours using survival analysis and Mann Whitney tests
4. We will compare the proportion of the first 48 hours spent in RASS -4 or -5 (based on hourly data recording) between the patients in the two groups using the Mann Whitney test
5. Adverse event rates during the first 48 hours will be compared

Secondary outcome measures

1. Duration of mechanical ventilation will be compared using survival analysis and Mann-Whitney test (using groups censored and uncensored for deaths)
2. ICU and hospital mortality will be compared between the groups
3. Total dose of sedative drug during the first 48 hours, and during ICU stay (up to seven days post randomisation) will be compared by Mann Whitney test
4. Mean sedation drug use per hour during the first 48 hours in ICU (or when first sedated and ventilated) and during ICU stay (up to seven days post randomisation) will be compared by Mann Whitney test
5. Total dose of opioid drug during the first 48 hours in ICU (or when first sedated and ventilated), and during ICU stay (up to seven days post randomisation) will be compared by Mann Whitney test
6. Mean opioid drug use per hour during the first 48 hours in ICU (or when first sedated and ventilated) and during ICU stay (up to seven days post randomisation) will be compared by Mann Whitney test

Quality control and data management

All CRF data will be entered into a database designed for the study. QC checks will be built in to the data base. In addition, we will undertake source data verification for a random sample of 10% of CRFs. Monitoring and data management will be undertaken at GE Healthcare, Helsinki.

Appendices

Appendix 1. Existing randomised trials of sedation monitoring in intensive care

Publication and type of study	Patient entry criteria and sample size	Intervention	Control	Results	Comments
[25] CCM 2008<br><br>Single centre unblinded RCT	Adult, mechanically ventilated<br><br>Sample size; intervention group n=153; control group n= 159	Patients received sedation directed by formal guidelines.	Patients received sedation by usual local clinical practice	No evidence of a substantial reduction in the duration of mechanical ventilation or length of stay, in either the intensive care unit or the hospital, with the use of protocol-directed sedation compared with usual local management.	I centre<br><br>High intensity staffing levels and nurse responsibility may be the cause of the contrasting results.
[24] Lancet 2008<br><br>Multicentre prospective unblended RCT	Mechanical ventilation >12 hours<br><br>Exclusions<br>Post cardiopulmonary arrest; mechanical	Spontaneous wakening trial plus spontaneous breathing trial following tight protocol	Individual unit standard protocols<br><br>Patient targeted sedation with titration to clinically appropriate	Reduction in ventilator-free days during 28 days follow-up (14.7 versus 11.6)<br><br>Higher conscious level at first successful SBT	4 centres<br>Only medical patients<br>No pre-defined frequency of sedation scoring in control group.<br>Investigators documented RASS twice daily<br>Both groups received SBTs at similar rates

	ventilation >14 days; moribund state; withdrawal of life support; profound neurological deficit<br><br>Sample size Intervention group 168 Control group 168		levels<br><br>Depth of sedation monitored using validated scale	Shorter duration of coma (RASS < -3; 2 versus 3 days)<br><br>Shorted ICU and hospital stays<br><br>Lower 1 years mortality	Major difference between groups was use of a spontaneous awakening trial (90 versus 0%)<br>Not all patients in coma on first study day (RASS <-3)<br>Time from admission to enrolment median 2.2 days<br>Time to first successfully passed SBT similar for both groups (intervention median 3.8 versus control 3.9 days)<br>Intervention group had more awake RASS at time of first successful SBT<br>More patients were extubated after their first SBT in the intervention than control groups (54 versus 40%
[23] CCM 2006<br><br>Prospective unblended RCT in two medical ICUs	Adult patients expected to require >48 hours of mechanical ventilation	Propofol by continuous infusion to achieve a Ramsay score of 2-3 plus daily	Intermittent lorazepam boluses to achieve a Ramsay score of 2-3 plus	Primary outcome was median ventilator days<br><br>Propofol group had shorter median	Lorazepam group had higher f/TV ratios during weaning trials suggesting an effect of lorazepam sedation on respiratory function.

	Sample size 64 (lorazepam) versus 68 (propofol)	interruption of sedation	daily interruption of sedation	duration of ventilation (5.8 versus 8.4, P = 0.4).<br><br>Survivors had shorter ICU length of stay.<br><br>No difference in hospital mortality	Propofol group were more likely to receive morphine infusions and had higher mean morphine use per ICU day
[34] CCM 2007 Prospective before and after study. Two 2-yrs long phases separated by a 6 months implementation phase	All adult patients requiring ≥48 hours mechanical ventilation receiving infusions with propofol or midazolam alone.<br><br>Exclusions<br>MV for >24hrs prior to ICU admission<br>Use of analgesics<br>Peripheral nervous system disorders<br>Admission after cardiac arrest	Nurse implemented sedation protocol<br><br>Cambridge score used to assess patients 3 hourly<br>Drug dosage closely controlled according to patient weight and responses.	No protocol used	Primary endpoint was incidence of VAP<br>Significant reduction in VAP incidence in intervention group (6% versus 15%, P = 0.005; 14.5 per 1000 ventilator days versus 19.3 per 1000 ventilator days, P = 0.45)<br><br>Duration of mechanical ventilation decreased<br>Median 8 versus 4.2 days	Single centre study<br><br>Carried out 1999 to 2003<br><br>No systematic change to weaning practice during intervention<br><br>Multivariate analysis showed protocol implementation strongly associated with reduction in VAP

	Sample size<br>Before intervention 226 analysed<br>After intervention 197 analysed			Decreased in time from ending sedative infusion to extubation and reduction in failed extubations<br>Lower daily doses of midazolam and propofol<br><br>Decreased length of stay in ICU (11 versus 5 days)<br>Decreased length of stay in hospital (21 versus 17 days)<br><br>Trend to lower hospital mortality (45% versus 38%; NS)	
[35]. Acta Anaesth Scand 2006<br><br>Before and after quality improvement project	Eligibility criteria not clearly stated<br><br><br><br>Sample size<br>Pre-intervention 166 patient days (24 patients)	Introduction of an SOP to achieve target Ramsay scores of 2/3 during day and 3/4 at night.<br><br>Regular	Control period similar goals and practice but no re-inforcement of SOP	Increased recording of Ramsay score following implementation 71% versus 62%<br><br>No change in Ramsay scores<br>No change in	Small single centre study<br><br>Shows that implementation problematic even with established sedation algorithms

	Post-intervention 170 patient days (29 patients)	measurement and recording of Ramsay score<br><br>A daily sedation break or "tapering"		ventilation or length of stay paramenters<br><br>No increase in sedation breaks	
[36]CCM 2006<br><br>Prospective before and after study (control phase 21 weeks; training phase 4 weeks; intervention phase 29 weeks)	All patients staying in ICU >24 hours<br><br>Exclusions<br>Readmission to ICU<br>Brain injury<br>Transfer to another ICU<br>Decision to withdraw treatment within 48 hours<br><br>Sample size<br>Pre-intervention 100 evaluated<br>Post-intervention 130 evaluated	Bedside nurse evaluated RASS and BPS 3 times daily and after any procedure<br><br>No specific protocols for drug management documented	No systematic evaluation of pain or agitation by nurses<br><br>Independent nurses assessed RASS and BPS twice daily blinded to cliical staff	Reduction in proportion of patients with pain and agitation (primary end point)<br>Pain: 63% versus 42%<br>Agitation: 29% versus12%<br><br>Marked decrease in mechanical ventilation<br>Median 120 to 65 hours<br><br>Reduction in all infection outcomes	Mixed medical surgical ICU<br>Single centre<br><br>Emphasis on detection and treatment of pain and agitation. Little emphasis on excessive sedation.<br><br>Duration of hypnotic infusion was decreased significantly (median 84 to 48 hours)
[37] CCM 2005	Adult patients	Sedation state	No sedation	Reduction in	Single centre study

Prospective before and after study separated by 9 month implementation phase	requiring ≥24 hours mechanical ventilation<br><br>Exclusions<br>Acute brain injury<br>Transfer from another ICU<br>Chronic mechanical ventilation<br>Peripheral nervous system disorder<br>Existing tracheostomy<br>Moribund state<br><br>Sample size<br>54 pre- and 48 post- intervention	assessed every 3 hours by ATICE score<br><br>Sedation followed algorithm based on ATICE score	algorithm used<br><br>Sedation controlled by physician.<br>Nurses not able to change without physician input	duration of mechanical ventilation<br>Median 4.4 versus 10.3 days<br><br>Reduction in ICU stay<br>Median 8.0 versus 15.0 days<br><br>Time to arousal shorter<br>Median 2 versus 4 days<br><br>Intervention group associated with:<br>Lower incidence pressure sores<br>Lower VAP rates (not statistically significant)<br>Lower cumulative and mean daily midazolam doses (but not duration of treatment	Study carried out 1999-2001<br>Mixed medical surgical ICU<br><br>No change in weaning practice across periods<br>Association between time to arousal and duration of mechanical ventialtion
[38]CCM 2003	Adult patients requiring >3 days	Multidisciplinary group devised	Control group data obtained	Reduction in Median MV days	Single hospital organisation-wide

Mixed retrospective/prospective before and after quality improvement project in 5 hospital ICUs	mechanical ventilation<br><br>No exclusions<br><br>Sample size<br>Pre-intervention 595 (18 months sample)<br>Post-intervention 520 (12 months sample size)	protocols and pathways for sedation and weaning. Little detail of exact practices implemented<br><br>Dedicated nurse specialists employed to implement and monitor.	through retrospective data extraction	(10 versus 9)<br>ICU length of stay 15 versus 10)<br>Hospital length of stay (22 versus 20)<br>Mortality 38% versus 31%	evaluation<br>Coronary care, surgical, medical, neurointensive care, and thoracic cardiovascular ICU
[39] BMJ 2002<br><br>Prospective quality improvement project	All adult patients mechanically ventilated >24 hours<br><br>No stated exclusions<br><br>Sample size<br>147 pre- and 138 post- intervention	Doctors defined level of sedation desired twice per day. Nurse used sedation scoring system to adjust drugs to achieve desired level.<br><br>No daily wake up or predefined weaning practice<br><br>Pre-intervention and post-	Not applicable	Using statistical process control methodology mean ventilator time decreased by 28% (7.4 to 5.3 days<br><br>Trend towards lower length of stay in ICU (9.3 versus 8.3 days)<br><br>Trend towards lower mortality post intervention (27% versus 22% (difference 5% (95%	Single centre study<br>Surgical patients<br>Study carried out 1999<br><br>Uncontrolled study<br>No pre-defined frequency of sedation scoring<br><br>No excess adverse events reported but uncontrolled

		intervention periods of 11 months		CI –0.5 to 14.9%)	
[21] NEJM 2000<br><br>Single centre prospective unblended RCT	Mechanically ventilated patients requiring continuous sedation by infusion<br><br>Exclusions<br>Post cardiac arrest<br>Transfers from another institution with sedation already established<br><br>Sample size<br>Intervention group 68<br>Control group 60	Daily interruption of sedation starting 48 hours after enrolment until awake and following instructions or uncomfortable and/or agitated requiring re-sedation.<br><br>Review by a physician once the patient was awake to determine further management	Adjustment of sedation at discretion of intensive care unit team	Shorter duration of mechanical ventilation (4.8 versus 7.3 days<br><br>Percent days during which patients were awake dramatically different (85.5% versus 9%)<br><br>Shorter ICU stay<br><br>No difference in hospital stay<br><br>For propofol subgroup no difference in dose<br>For midazolam subgroup lower doses in intervention group<br><br>Trend towards higher survival rates and rates of	Single centre study<br>Medical patients<br>No pre-defined use or frequency of scoring system<br>No long term outcomes<br>No standardisation of weaning<br><br>Subsequent analyses showed lower rates of PTSD and better adjustment to illness after discharge in intervention groups

				discharge to own home (neither statistically significant)<br><br>No excess of adverse events<br>Fewer diagnostic tests to investigate coma in intervention group	
[31] CCM 1999<br><br>Prospective unblinded RCT	All admissions to medical ICU<br><br>Sample size<br>Intervention 162<br>Control 159	Patients managed by nursing staff using an algorithm<br><br>Target sedation score Ramsay 3	Sedation controlled by physician orders	Intervention group showed :<br>Reduction in mechanical ventilation duration (89 versus 124 hours)<br>Reduction in ICU stay (5.7 versus 7.5 days)<br>Hospital stay (14.0 versus 19.9 days)<br><br>Tracheostomy rate	Medical patients<br>High use of benzodiazepines<br><br>Control group probably not relevant to contemporary practice for most centres (physician orders)<br><br>Weaning practice not controlled

Appendix 2: Published studies and guidelines relating to sedation practice in ICUs. Recommendations relating to frequency of use of clinical sedation scales.

Author, Publication & Year of Publication	Title of Paper	Type of study	Recommended Sedation Score Frequency	Comments
Royal College of Anaesthetists	Implementing & ensuring Safe Sedation Practice for healthcare procedures in adults	Working Party Report	Nil	One team member should have responsibility for patient observation and record keeping
[40] Paediatric Nursing (2003)	Guidelines for sedation of the critically ill child	Practice review	Nil	A desired sedation score should be documented by medical staff
[39] BMJ (2002)	Effect of a scoring system and protocol for sedation on duration of patients' need for ventilator support in a surgical ICU	Observational study	Nil	Medical staff defined level of sedation twice a day and nurse in charge of pt was responsible for monitoring and adjusting sedation as per guidance
Intensive Care society	Sedation Guideline	Guideline	Nil	Sedation should be managed precisely and given priority attention
European Association for Palliative Care	Monitoring of sedation at end of life, supportive care for families	Literature review	Nil	Level of sedation should be assessed daily and reviewed in light of patient goals

American Association of Nurse Anaesthetists	Considerations for policy Guidelines for Registered Nurses Engaged in the Administration of Sedation and Analgesia	Guidelines	Nil	
[27] Critical Care (2007)	Changes in sedation management in German ICUs between 2002 & 2006: national follow-up survey	Follow-up survey with descriptive & comparative cross-sectional multi-center design	Nil	There was an increased use in sedation scoring between the 2 years
[32] Critical Care Medicine (2002)	Clinical practice guidelines for the sustained use of sedatives & analgesics in the critically ill adult	Discussion	Regular	A sedation goal endpoint should be established and regularly redefined for each patient. Regular assessment & response to therapy should be systematically documented
[41] Critical Care Medicine (2005)	Large-scale implementation of sedation & delirium monitoring in the ICU: A report from two medical centers	Prospective observational cohort study	4 hourly (3 times per 12hr shift)	
[42] Critical Care Medicine (2005)	ICU delirium is an independent predictor of	A prospective cohort	Once per 12 hour shift	This was the study recording not nursing

	longer hospital stay: a prospective analysis of 261 non-ventilated patients			recording
[43] The American Journal of geriatric pharmacotherapy (2007)	Clinical sedation scores as indicators of sedative and analgesics drug exposure in ICU patients	Prospective observational pilot study	Twice daily	
[44] Anaesthesia (2006)	Impact of introducing a sedation management guideline in ICU	Prospective observational study	Hourly	
[8] JAMA (2003)	Monitoring Sedation Status Over Time in ICU Patients	Prospective cohort study	Nil	Only a single RASS score per pt assessment was taken for study purposes
[45] Advanced Critical Care (2007)	Perspectives on sedation assessments in critical care	Summary of sedation scales	Nil	Frequent stimulation for sedation assessment would interfere with sleep and recovery
[46] The Annals of Pharmacotherapy (2004)	Sedation Assessment in Critically Ill Adults: 2001-2004 Update	Literature Review	Nil	Goal driven sedation therapy improves patient outcomes 7 there are several useful tools

				available to guide sedation therapy
[5] Am J Respir Crit Care Med (2002)	The Richmond Agitation-Sedation Scale: Validity & Reliability in Adult ICU Patients		Nil	
[47] Journal of Advanced Nursing (2006)	Adoption of a sedation scoring system & sedation guideline in an ICU	Quasi-experimental pre intervention & post intervention quality improvement design	4 hourly documentation	

Reference List

1. Sessler CN. Sedation scales in the ICU. Chest 2004;126(6):1727-30.

2. Ramsay MAE, Savege TM, Simpson BRJ, Goodwin R. Controlled Sedation with Alphaxalone-Alphadolone. British Medical Journal 1974;2(5920):656-9.

3. Riker RR, Picard JT, Fraser GL. Prospective evaluation of the Sedation-Agitation Scale for adult critically ill patients. Critical Care Medicine 1999;27(7):1325-9.

4. Devlin JW, Boleski G, Mlynarek M, Nerenz DR, Peterson E, Jankowski M, Horst HM, Zarowitz BJ. Motor Activity Assessment Scale: A valid and reliable sedation scale for use with mechanically ventilated patients in an adult surgical intensive care unit. Critical Care Medicine 1999;27(7):1271-5.

5. Sessler CN, Gosnell MS, Grap MJ, Brophy GM, O'Neal PV, Keane KA, Tesoro EP, Elswick RK. The Richmond Agitation-Sedation Scale - Validity and reliability in adult intensive care unit patients. American Journal of Respiratory and Critical Care Medicine 2002;166(10):1338-44.

6. De Jonghe B, Cook D, Griffith L, ppere-De-Vecchi C, Guyatt G, Theron V, Vagnerre A, Outin H. Adaptation to the Intensive Care Environment (ATICE): Development and validation of a new sedation assessment instrument. Critical Care Medicine 2003;31(9):2344-54.

7. Weinert C, McFarland L. The state of intubated ICU patients - Development of a two-dimensional sedation rating scale for critically ill adults. Chest 2004;126(6):1883-90.

8. Ely EW, Truman B, Shintani A, Thomason JWW, Wheeler AP, Gordon S, Francis J, Speroff T, Gautam S, Margolin R, et al. Monitoring sedation status over time in ICU patients - Reliability and validity of the Richmond Agitation-Sedation Scale (RASS). Jama-Journal of the American Medical Association 2003;289(22):2983-91.

9. Punjasawadwong Y, Boonjeungmonkol N, Phongchiewboon A. Bispectral index for improving anaesthetic delivery and postoperative recovery. Cochrane Database of Systematic Reviews 2007;(4).

10. Myles PS, Leslie K, McNeil J, Forbes A, Chan MTV. Bispectral index monitoring to prevent awareness during anaesthesia: the B-Aware randomised controlled trial. Lancet 2004;363(9423):1757-63.

11. Avidan MS, Zhang LN, Burnside BA, Finkel KJ, Searleman AC, Selvidge JA, Saager L, Turner MS, Rao S, Bottros M, et al. Anesthesia awareness

and the bispectral index. New England Journal of Medicine 2008;358(11):1097-108.

12. Nasraway SA. The Bispectral Index: Expanded performance for everyday use in the intensive care unit? Critical Care Medicine 2005;33(3):685-7.

13. Ely EW, Truman B, Manzi DJ, Sigl JC, Shintani A, Bernard GR. Consciousness monitoring in ventilated patients: bispectral EEG monitors arousal not delirium. Intensive Care Medicine 2004;30(8):1537-43.

14. Tonner PH, Wei C, Bein B, Weiler N, Paris A, Scholz J. Comparison of two bispectral index algorithms in monitoring sedation in postoperative intensive care patients. Critical Care Medicine 2005;33(3):580-4.

15. Vivien B, Di Maria S, Ouattara A, Langeron O, Coriat P, Riou B. Overestimation of bispectral index in sedated intensive care unit patients revealed by administration of muscle relaxant. Anesthesiology 2003;99(1):9-17.

16. Weatherburn C, Endacott R, Tynan P, Bailey M. The impact of Bispectral Index monitoring on sedation administration in mechanically ventilated patients. Anaesthesia and Intensive Care 2007;35(2):204-8.

17. Mehta S, Burry L, Fischer S, Martinez-Motta JC, Hallett D, Bowman D, Wong C, Meade MO, Stewart TE, Cook DJ. Canadian survey of the use of sedatives, analgesics, and neuromuscular blocking agents in critically ill patients. Critical Care Medicine 2006;34(2):374-80.

18. Walsh TS, Ramsay P, Lapinlampi TP, Sarkela MOK, Viertio-Oja HE, Merilainen PT. An assessment of the validity of spectral entropy as a measure of sedation statein mechanically ventilated critically ill patients. Intensive Care Medicine 2008;34(2):308-15.

19. Walsh TS, Ramsay P, Kinnunen R. Monitoring sedation in the intensive care unit: can "black boxes" help us? Intensive Care Medicine 2004;30(8):1511-3.

20. Kress JP, Gehlbach B, Lacy M, Pliskin N, Pohlman AS, Hall JB. The long-term psychological effects of daily sedative interruption on critically ill patients. American Journal of Respiratory and Critical Care Medicine 2003;168(12):1457-61.

21. Kress JP, Pohlman AS, O'Connor MF, Hall JB. Daily interruption of sedative infusions in critically ill patients undergoing mechanical ventilation. New England Journal of Medicine 2000;342(20):1471-7.

22. Kress JP, Vinayak AG, Levitt J, Schweickert WD, Gehlbach BK, Zimmerman F, Pohlman AS, Hall JB. Daily sedative interruption in mechanically ventilated patients at risk for coronary artery disease. Critical Care Medicine 2006;35(2):365-71.

23. Carson SS, Kress JP, Rodgers JE, Vinayak A, Campbell-Bright S, Levitt J, Bourdet S, Ivanova A, Henderson AG, Pohlman A, et al. A randomized trial of intermittent lorazepam versus propofol with daily interruption in mechanically ventilated patients. Critical Care Medicine 2006;34(5):1326-32.

24. Girard TD, Kress JP, Fuchs BD, Thomason JWW, Schweickert WD, Pun BT, Taichman DB, Dunn JG, Pohlman AS, Kinniry PA, et al. Efficacy and safety of a paired sedation and ventilator weaning protocol for mechanically ventilated patients in intensive care (Awakening and Breathing Controlled trial): a randomised controlled trial. Lancet 2008;371(9607):126-34.

25. Bucknall TK, Manias E, Presneill JJ. A randomized trial of pyotocol-directed sedation management for mechanical ventilation in an Australian intensive care unit. Critical Care Medicine 2008;36(5):1444-50.

26. Devlin JW, Tanios MA, Epstein SK. Intensive care unit sedation: Waking up clinicians to the gap between research and practice. Critical Care Medicine 2006;34(2):556-7.

27. Martin J, Franck M, Sigel S, Weiss M, Spies C. Changes in sedation management in German intensive care units between 2002 and 2006: a national follow-up survey. Critical Care 2007;11(6).

28. Christensen BV, Thunedborg LP. Use of sedatives, analgesics and neuromuscular blocking agents in Danish ICUs 1996/97 - A national survey. Intensive Care Medicine 1999;25(2):186-91.

29. Samuelson KA, Larsson S, Lundberg D, Fridlund B. Intensive care sedation of mechanically ventilated patients: a national Swedish survey. Intensive Crit Care Nurs 2003;19(6).

30. Payen JF, Chanques G, Mantz J, Hercule C, Auriant I, Leguillou JL, Binhas M, Genty C, Rolland C, Bosson JL. Current practices in sedation and analgesia for mechanically ventilated critically ill patients - A prospective multicenter patient-based study. Anesthesiology 2007;106(4):687-95.

31. Brook AD, Ahrens TS, Schaiff R, Prentice D, Sherman G, Shannon W, Kollef MH. Effect of a nursing-implemented sedation protocol on the duration of mechanical ventilation. Critical Care Medicine 1999;27(12):2609-15.

32. Jacobi J, Fraser GL, Coursin DB, Riker RR, Fontaine D, Wittbrodt ET, Chalfin DB, Masica MF, Bjerke HS, Coplin WM, et al. Clinical practice guidelines for the sustained use of sedatives and analgesics in the critically ill adult. Critical Care Medicine 2002;30(1):119-41.

33. Dellinger RP, Levy MM, Carlet JM, Bion J, Parker MM, Jaeschke R, Reinhart K, Angus DC, Brun-Buisson C, Beale R, et al. Surviving Sepsis Campaign: International guidelines for management of severe sepsis and septic shock: 2008. Critical Care Medicine 2008;36(1):296-327.

34. Quenot JP, Ladoire S, Devoucoux F, Doise JM, Cailliod R, Cunin N, Aube H, Bleftery B, Charles PE. Effect of a nurse-implemented sedation protocol on the incidence of ventilator-associated pneumonia. Critical Care Medicine 2007;35(9):2031-6.

35. Tallgren M, Pettila V, Hynninen M. Quality assessment of sedation in intensive care. Acta Anaesthesiologica Scandinavica 2006;50(8):942-6.

36. Chanques G, Jaber S, Barbotte E, Violet S, Sebbane M, Perrigault PF, Mann C, Lefrant JY, Eledjam JJ. Impact of systematic evaluation of pain and agitation in an intensive care unit. Critical Care Medicine 2006;34(6):1691-9.

37. De Jonghe B, Bastuji-Garin S, Fangio P, Lacherade JC, Jabot J, ppere-De-Vecchi C, Rocha N, Outin H. Sedation algorithm in critically ill patients without acute brain injury. Critical Care Medicine 2005;33(1):120-7.

38. Burns SM, Earven S, Fisher C, Lewis R, Merrell P, Schubart JR, Truwit JD, Bleck TP. Implementation of an institutional program to improve clinical and financial outcomes of mechanically ventilated patients: One-year outcomes and lessons learned. Critical Care Medicine 2003;31(12):2752-63.

39. Brattebo G, Hofoss D, Flaatten H, Muri AK, Gjerde S, Plsek PE. Quality improvement report - Effect of a scoring system and protocol for sedation on duration of patients' need for ventilator support in a surgical intensive care unit. British Medical Journal 2002;324(7350):1386-9.

40. Bennett M. Guidelines for sedation of the critically ill child. Paediatr Nurs 2003;15(9).

41. Pun BT, Gordon SM, Peterson JF, Shintani AK, Jackson JC, Foss J, Harding SD, Bernard GR, Dittus RS, Ely EW. Large-scale implementation of sedation and delirium monitoring in the intensive care unit: A report from two medical centers. Critical Care Medicine 2005;33(6):1199-205.

42. Thomason JWW, Shintani A, Peterson JF, Pun BT, Jackson JC, Ely EW. Intensive care unit delirium is an independent predictor of longer hospital stay: a prospective analysis of 261 non-ventilated patients. Critical Care 2005;9(4):R375-R381.

43. Masica AL, Girard TD, Wilkinson GR, Thomason JWW, Truman Pun B, Nair UB, Light RW, Canonico AE, Dunn J, Pandharipande P, et al. Clinical sedation scores as indicators of sedative and analgesic drug

exposure in intensive care unit patients. Am J Geriatr Pharmacother 2007;5(3).

44. Adam C, Rosser D, Manji M. Impact of introducing a sedation management guideline in intensive care. Anaesthesia 2006 Mar;61(3):260-3.

45. Olson DM, Thoyre SM, Auyong DB. Perspectives on sedation assessment in critical care. AACN Adv Crit Care 2007;18(4).

46. Watson BD, Kane-Gill SL. Sedation assessment in critically ill adults: 2001-2004 update. Annals of Pharmacotherapy 2004;38(11):1898-906.

47. Elliott R, McKinley S, Aitken L. Adoption of a sedation scoring system and sedation guideline in an intensive care unit. Journal of Advanced Nursing 2006;54(2):208-16.
