# Supplementary material for: A randomized controlled proof-of-concept trial of early sedation management using Responsiveness Index monitoring in mechanically ventilated critically ill patients
Source: Crit Care. 2015 Sep 11;19(1):333. doi: 10.1186/s13054-015-1043-1 (PMC4570737; doi:10.1186/s13054-015-1043-1)
Supplement: Additional file 2: — Summary of qualitative analysis of interviews with ICU nurses regarding their views and opinions of responsiveness index monitoring in the IMPROVE pilot trial. (PDF 288 kb) [file 13054_2015_1043_MOESM2_ESM.pdf]

Summary of Qualitative analysis of interviews with ICU nurses regarding their views and opinions of Responsiveness Index monitoring in the trial.

Methodology/Design

The study was approved by the local ethics committee. Participants were identified using purposeful sampling; all nurses approached agreed to participate. Eight male and eight female nurses were interviewed; ICU nursing experience ranged from 3 months to 18 years. All participants had a minimum of 12 hours experience using the RM. An aide memoir was used to prompt the flow of the interviews. All interviews took place while the nurses were at the bedside at a time that was convenient for them and did not impact upon patient care. A number of the interviews took place while the RM was in use. This context proved very important, allowing the nurses to point and refer to real, and occurring clinical situations to complement and support their narratives, and captured relevant non-verbal gestures. Interviews lasted between 20 and 45 minutes and were digitally recorded for later analysis; in addition, the researcher made field notes immediately following the interview, noting non-verbal body language used by the participant.

Theoretical perspective

The research design was based on interpretivism, and underpinned by a phenomenological approach. This was chosen because the focus was on human experience as lived by the participants in relation to sedation practices, and specifically the use of the RM. In keeping with Heideggerian phenomenology the researcher (KE) acknowledged her presuppositions and their potential bearing on interpretation before commencing the interviews.

Interviews focussed on the changes participants had experienced in ICU sedation management and the use of technologies in ICU. This specifically included the usability and acceptability of the responsiveness monitor (RM) in their daily practice. This work formed part of a PhD thesis: *"Phenomenological exploration of clinical decision making of Intensive Care Unit (ICU) nurses in relation to sedation management"*, which is accessible in full online (www.era.lib.ed.ac.uk/handle/1842/7773).

The researcher adapted Van Manen's framework to guide the analysis process [1]. The early interview narratives were quickly reinforced in subsequent interviews unveiling potent commonalities in the nurses' perceptions, understandings and experiences.

Findings

General findings:

Important findings of the general exploration of views in relation to novel approaches to sedation included unforeseen consequences from well-intentioned initiatives to improve the quality of patients' care, especially the use of sedation breaks/holds and maintaining greater wakefulness. The ICU nurses described the current 'target' approach to changes in sedation management as a threat to their professional obligation and personal desire to provide holistic care; it ran counter to the individualised care they want to deliver. Patients' sedation status also directly impacted upon the nurses' workload and left them in a state of disequilibrium regarding the requirement to deliver evidence-based care (greater wakefulness and sedation holds), the desire to deliver holistic care, and the duty to deliver safe care (especially avoiding sedation-related adverse events). Key themes of approaches to minimise the use of sedation were therefore 'working

priorities' and 'unintended consequences'. The use of lighted sedation states, and strategies to achieve this, often left the ICU nurses in fear for their patient's safety despite these interventions being aimed at increasing patient safety and wellbeing. This also generated anxiety and tension for nurses themselves. These findings have been reported separately [2].

Responsiveness monitoring (RM):

Participants considered the RM and its influence on, and implications for, their nursing practice and decisions. The RM was thought to potentially alleviate some of the fear of the 'unknown' associated with a more wakeful ICU population, and offered the nurses confidence and supported their professional autonomy.

In practical terms, the RM was welcomed as a non-invasive monitoring device, designed with trends and numbers in familiar formats, identifiable colours and alarm-free:

"...they are unobtrusive... easy to set up, just stick the sensors on, plug the machine in, away you go..." (Interviewee 009)

"I think in intensive care we are used to having waveforms so I really like that but I think the thing I find most useful is the colour coding so that you can automatically, just from glancing at it, you can...have knowledge from...the colour the number is in....it seems quite user friendly... it is very similar to the waveforms we already have on the monitors... the waveform with a number at the end....it is nice and simple, it is not over complicated" (Interviewee 001)

"... it is very helpful because ... if the patient is lying in the red zone for no apparent reason...then it …concentrates your care that something has to be done..." (Interviewee 002)

"...I think you have this visual reminder and it's a bit more obvious to not just you but everybody passing by, so you think oh they're in a red zone, you would probably have to try and reduce that down" (Interviewee 014)

It dispelled subjectivity that was often associated with performing a sedation score and was able to provide a continuous and objective overview of patients; nurses didn't have to stop and discretely collect any information to ascertain patients' responsiveness level:

"I think it probably prompted you to think about it [sedation] more because you can also write an hourly RASS without much thought, so for me it probably prompted me to think 'yes well actually she is a –3', or whatever maybe I should be turning [sedation down]… it is a prompt..." (Interviewee 008)

"I think it is much more of a reminder...you know you don't necessarily do your RASS every single hour of your observations, yet I look at the monitor, I have been seeing the trace on the monitor a lot more than I would actually have done the RASS" (Interviewee 001)

"*Sometimes you can be so busy...that in as much as you would be looking just in a split second, as you can see, 28 and it will amaze you that within 5 minutes it has dropped into the red zone, so I think if you were very busy...if there was something just to alert you..."* (Interviewee 002)

The nurses also suggested that the RM helped make the invisible 'visible':

"I think it is much more of a reminder...I have been seeing the trace on the monitor a lot more than I would actually have done the RASS" (Interviewee 001)

"...it's maybe a bit more obvious to not just you but everybody passing by, so you think oh they're in a red zone, you would probably have to try and reduce that down...a warning in your face...it's less easier to ignore it..." (Interviewee 014)

It was perceived by the nurses that the medical staff would not believe them that their patient had been agitated and the RM could provide the visible objective proof:

"...well there you are there's your proof...you could say look even on this sedation they've been fairly restless....." (Interviewee 008)

The RM also provided advance warning of often unpredictable waking of a patient which they openly expressed they feared, especially in relation to the use of sedation holds and reductions (frequently dictated by medical staff).

"I just think that people waking up is one of the hardest, one of the hardest things we have to witness here, because people are uncomfortable, they get a fright, you know it is quite nerve racking sometimes because you don't know what is going to happen and I think anything that is preparing you more to be prepared for things is good" (Interviewee 004)

"It's a guide...it gives you a bit more confidence in what you're doing in terms of lightening the patient or maybe giving them more sedation, it help you decide what to do I think…It confirmed what I was thinking so I felt more comfortable then in reducing it a bit" (Interviewee 005)

Interpretation

From the perceptions revealed by the nurses, regardless of experience and expertise, the RM potentially offered a way of addressing the challenges wakefulness posed and the potent

emotions of fear, loss of control and confidence experienced in the ICU nurses' 'world'. However, caution was expressed in the way in which it might be used. If the monitor is used to 'dictate' ranges of responsiveness or achieve specific targets, especially if driven by medical staff, nurses perceived the value of the monitor's information might shift from a tool to *assist* decision-making to another potential source of conflict. The monitor was perceived as having greatest value if used to support nursing decision-making while maintaining autonomy, rather than as a target set by medical staff. A potential dilemma exists whereby whilst simultaneously increasing awareness of sedation status in a non-invasive manner the RM could become another ICU technology to divert the nurse's focus away from the patient him/herself. Nurses generally recalled its use in a favourable manner, but as an *adjunct* to the care they provided. Given the meaning of RI values are different from many physiological variable used in the ICU, for example a red value can occur from excessive deep sedation, illness-related coma, sleep, or pharmacologic neuromuscular paralysis, effective use requires adequate training and education. There were some instances when the information the RM displayed was not considered indicative of the patient's *actual* sedation state, potentially as a result of incomplete or inadequate understanding of the RI concept.

References

[1] Van Manen M (1990) Researching Lived Experience: Human Science for an Action Sensitive Pedagogy. The University of Western Ontario, London, ON.

[2] Everingham K. Fawcett T. Walsh T. Targeting' sedation: the lived experience of the intensive care nurse. Journal of Clinical Nursing. 23(5-6):694-703, 2014
